# Quality indicators for breast reconstruction following cancer—an international Delphi consensus study supported by the European Society of Plastic, Reconstructive and Aesthetic Surgery

**DOI:** 10.1093/bjsopen/zraf144

**Published:** 2025-11-24

**Authors:** Emma Hansson, Nicholas Moellhoff, Susanne Ahlstedt Karlsson, Alexandra Uusimäki, Ilkka Kaartinen, Lisbet Rosenkrantz Hölmich, Rado Zic, Ruth Waters, Mark Henley, Riccardo E Giunta, Anna Elander, the ESPRAS European Quality Indicator Group, Tanja Aaby Orellana, João Correia Anacleto, Andreas Arkudas, Giovanni Di Benedetto, Couturaud Benoit, Linda Bull Madsen, Burcu Celet Ozden, Giovanni Di Benedetto, Ann-Charlott Docherty Skogh, Tine Engberg Damsgaard, Patricia García Marcos, Ansis Gilis, Adam Gilmour, C Guerrero Paez, Moustapha Hamdi, Yves Harder, Cristian Radu Jecan, Ulla Karhunen-Enckell, Christian Korvald, Francisco Leyva, Daniel Maliszewski, Maria Mani, Päivi Merkkola-von Schantz, Camilla Morken Kristoffersen, Xavier Nelissen, Laura Pandis, Nikolaos A Papadopulos, Sari Pieper, Hinne Rakhorst, Maurizio Ressa, Marzia Salgarello, Fabio Santanelli di Pompeo, Kavita Sharma, Thomas Sjøberg, Karen Sundbøll, Cristina Triviño de la Cal, Maja Erceg Tušek, Ruth Waters, Carlos Villar Yus, Dolores Wolfram

**Affiliations:** Department of Plastic Surgery, Institute of Clinical Sciences, Sahlgrenska Academy, University of Gothenburg, Gothenburg, Sweden; Department of Plastic Surgery, Sahlgrenska University Hospital, Gothenburg, Sweden; Division of Hand, Plastic and Aesthetic Surgery, University Hospital LMU Munich, Munich, Germany; Department of Plastic Surgery, Institute of Clinical Sciences, Sahlgrenska Academy, University of Gothenburg, Gothenburg, Sweden; Department of Plastic Surgery, Sahlgrenska University Hospital, Gothenburg, Sweden; Institute of Health and Care Sciences, Sahlgrenska Academy, University of Gothenburg, Gothenburg, Sweden; Johanna, Regional branch of the Swedish Breast Cancer Association, Gothenburg, Sweden; Department of Musculoskeletal Surgery and Diseases, Clinic of Plastic and Reconstructive Surgery, Tampere University Hospital, Pirkanmaa Hospital District, Tampere, Finland; Department of Plastic Surgery, Copenhagen University Hospital, Copenhagen, Denmark; Department of Plastic, Reconstructive, and Aesthetic Surgery, University Hospital Dubrava, Zagreb, Croatia; Queen Elizabeth Hospital, University Hospitals Birmingham NHS Foundation Trust, Birmingham, UK; Department of Plastic and Reconstructive Surgery, Nottingham University Hospitals NHS Trust, Nottingham, UK; Division of Hand, Plastic and Aesthetic Surgery, University Hospital LMU Munich, Munich, Germany; Department of Plastic Surgery, Institute of Clinical Sciences, Sahlgrenska Academy, University of Gothenburg, Gothenburg, Sweden; Department of Plastic Surgery, Sahlgrenska University Hospital, Gothenburg, Sweden

**Keywords:** plastic surgery, surgical outcomes, patient-reported outcomes and experiences

## Abstract

**Background:**

Quality indicators (QIs) are essential for assessing and improving healthcare delivery. Existing QIs for breast reconstruction are limited and do not comprehensively reflect clinical complexity or patient-centred outcomes. This study aimed to develop a scientifically grounded, consensus-based set of QIs for breast reconstruction using the Delphi method.

**Methods:**

A structured Delphi process was conducted. Experts, including plastic surgeons, reconstructive nurses, and patient representatives from 21 European countries, were nominated by national professional and patient organizations. A pre-round generated 141 unique QIs, thematically analysed and categorized into six domains. Three Delphi rounds were conducted via electronic surveys. Consensus was defined *a priori* as ≥ 75% agreement across the whole group or at least two subgroups. Indicators were classified according to Donabedian’s model (structure, process, outcome).

**Results:**

Among the 43 experts completing all rounds, 41 QIs reached final consensus. These indicators span six key quality domains (Safety, Timeliness, Effectiveness, Efficiency, Equity, and Patient-centredness) and include measures such as access to reconstruction, treatment timelines, multidisciplinary collaboration, unit characteristics, surgical outcomes, and patient satisfaction. Structure, process, and outcome indicators were all represented, including patient-reported outcomes and patient-reported experiences.

**Conclusion:**

This Delphi study provides the first comprehensive set of QIs specific to breast reconstruction in Europe. These indicators lay the groundwork for future standardization, benchmarking, and quality improvement initiatives. Further work is needed to operationalize the indicators through evidence grading, measurement specifications, risk adjustment, and integration into clinical practice.

## Teaser

This study used a structured Delphi process involving experts and patient representatives from 21 European countries to develop a consensus-based set of 41 quality indicators for breast reconstruction. Spanning six quality domains and incorporating structure, process, and outcome measures, including patient-reported outcomes and patient-reported experiences, these quality indicators offer a comprehensive foundation for future benchmarking and quality improvement across Europe.

## Introduction

The World Health Organization (WHO) defines quality in healthcare as ‘the degree to which health services for individuals and populations increase the likelihood of desired health outcomes and are consistent with evidence-based professional knowledge’^1^. This concept encompasses six key domains, namely safety, timeliness, effectiveness, efficiency, equity, and patient-centredness, which together form the foundation for quality improvement frameworks^2^. Over recent decades, quality indicators (also known as performance measures) aligned with these domains have become important reporting and quality assurance tools to make performance data publicly available^3^. Their purposes include external accountability, performance verification, and supporting continuous improvement efforts^3^. Based on Donabedian’s model^2,4^, quality indicators fall into three categories, namely structure, process, and outcome, capturing the healthcare setting, the care delivered, and results for patients, respectively^3,5^. Each type of indicator has strengths and limitations, and a combined approach is often necessary for a comprehensive evaluation of quality^3^.

Effective quality indicators must be relevant, scientifically sound, feasible, and meaningful^3^. Ideally grounded in scientific evidence, they often also require expert input, particularly when evidence is lacking. Transparency in the development process and the composition of the expert group is essential for ensuring indicator validity^6^. Establishing an analytical framework is also critical to guide evidence gathering and evaluation, as well as for the rationale for the resulting quality indicators^7^. Narrowly focused indicators risk promoting measurement fixation^8^, where meeting targets supersedes delivering holistic care. For instance, clinicians may feel pressured to offer breast reconstruction to unsuitable candidates to satisfy performance metrics^8^. This narrow focus can lead to tunnel vision^8^, where unmeasured but important aspects of care, such as the risk of implant loss, long-term reconstructive outcomes, or revision rates, are neglected. Otherwise, there is a risk of gaming^8^, manipulating care decisions to enhance performance scores without genuinely improving care. Because breast reconstruction is a preference-sensitive intervention, indicators should reflect patient access and informed choice.

Currently, only two European disease-specific quality indicators exist for breast reconstruction, both developed by the European Society of Breast Cancer Specialists (EUSOMA) and included among 56 broader indicators for breast cancer care^9^. The indicators are the proportion of patients receiving immediate reconstruction at mastectomy (target: 60%; minimum: 40%) and the proportion of patients receiving immediate reconstruction while undergoing post-mastectomy radiotherapy (target: 40%; minimum: 20%)^9^. Although comprehensive in scope, these indicators fall short of adequately capturing all aspects of quality in breast reconstruction. Despite these efforts, it remains unclear whether current reconstruction indicators meaningfully improve care or outcomes^10^. Their methodological foundations, including how evidence was gathered and evaluated, and how standards were set, are insufficiently detailed^11,12^. In addition, the indicators fail to consider clinical factors, such as co-morbidities, that may contraindicate reconstruction or influence treatment decisions, which can vary by region or hospital type^5,12^. In brief, effective quality indicators must account for both positive outcomes (such as improved quality of life) and adverse outcomes (such as complications or revision operations) to promote responsible risk management and meaningful benefits in breast reconstruction.

In addition to the previously developed quality indicators, two relevant frameworks discuss quality in breast reconstruction. These frameworks are the International Consortium for Health Outcomes Measurement (ICHOM)^13^ initiative and the Breast Reconstruction And Valid Outcomes (BRAVO) core outcome set for breast reconstruction^14^. ICHOM^13^ emphasises patient-reported outcomes and complication rates (for example, Clavien–Dindo classification) for all breast cancer patients. The BRAVO core outcome set^14^ identifies outcomes important to patients and clinicians, such as major complications, quality of life, cosmetic satisfaction, and psychological wellbeing^14^. Although the two frameworks are useful in quality development in general, they did not specifically develop quality indicators.

To date, no scientifically validated quality indicators for breast reconstruction fully meet standards for credibility, validity, reliability, sensitivity, specificity, and feasibility^2,8,10^. To address this gap, the present study aimed to develop a new set of quality indicators using the Delphi technique.

## Methods

### Study protocol and ethics

This is the first study described in the Quality Indicators for Breast Reconstruction protocol (pre-registration: Region Västra Götaland Researchweb 284372; https://www.researchweb.org/is/vgr/project/284372). The study is reported according to the DELPHISTAR reporting guidelines^15^, and the DELPHISTAR checklist can be found in *Appendix S1*. The Declaration of Helsinki was followed during the procedures. All experts provided written informed consent for study participation and data management. The only data collected during the study were information on the expert category, the country represented, an e-mail address of choice (to enable reminders), suggestions for quality indicators, and grading of quality indicators. Names were not collected, and the experts could choose an e-mail address that did not reveal their identity. Nominations of experts were received by e-mail but were not registered. Hence, no sensitive data were registered. The European Society of Plastic Reconstructive and Aesthetic Surgery (ESPRAS) initiated and supported the study.

### Steering committee

The study’s steering committee is provided in *Appendix S2*.

### Experts

To represent the science and clinical practice of breast reconstruction, experts include plastic surgeons (the only surgical speciality performing all types of breast reconstruction), reconstructive nurses, and patient representatives. Experts had to fulfil predetermined criteria (*Table 1*) and be able to communicate in English.

**Table 1:** Study definitions of experts

Type of expert	Study definition
Plastic surgeon	Board-certified plastic surgeon in the country where they have worked for > 5 years Subspecialist in breast reconstruction > 5 years Works in a department of plastic surgery in a university hospital
Reconstructive nurse	Registered nurse in the country where they work Has worked regularly with breast reconstruction patients for > 3 years Works in a department of plastic surgery in a university hospital
Patient representative	Representative chosen by the elected board of a national or local patient organization for breast cancer patients

### Included countries and the number of experts

**Fig. 1:**
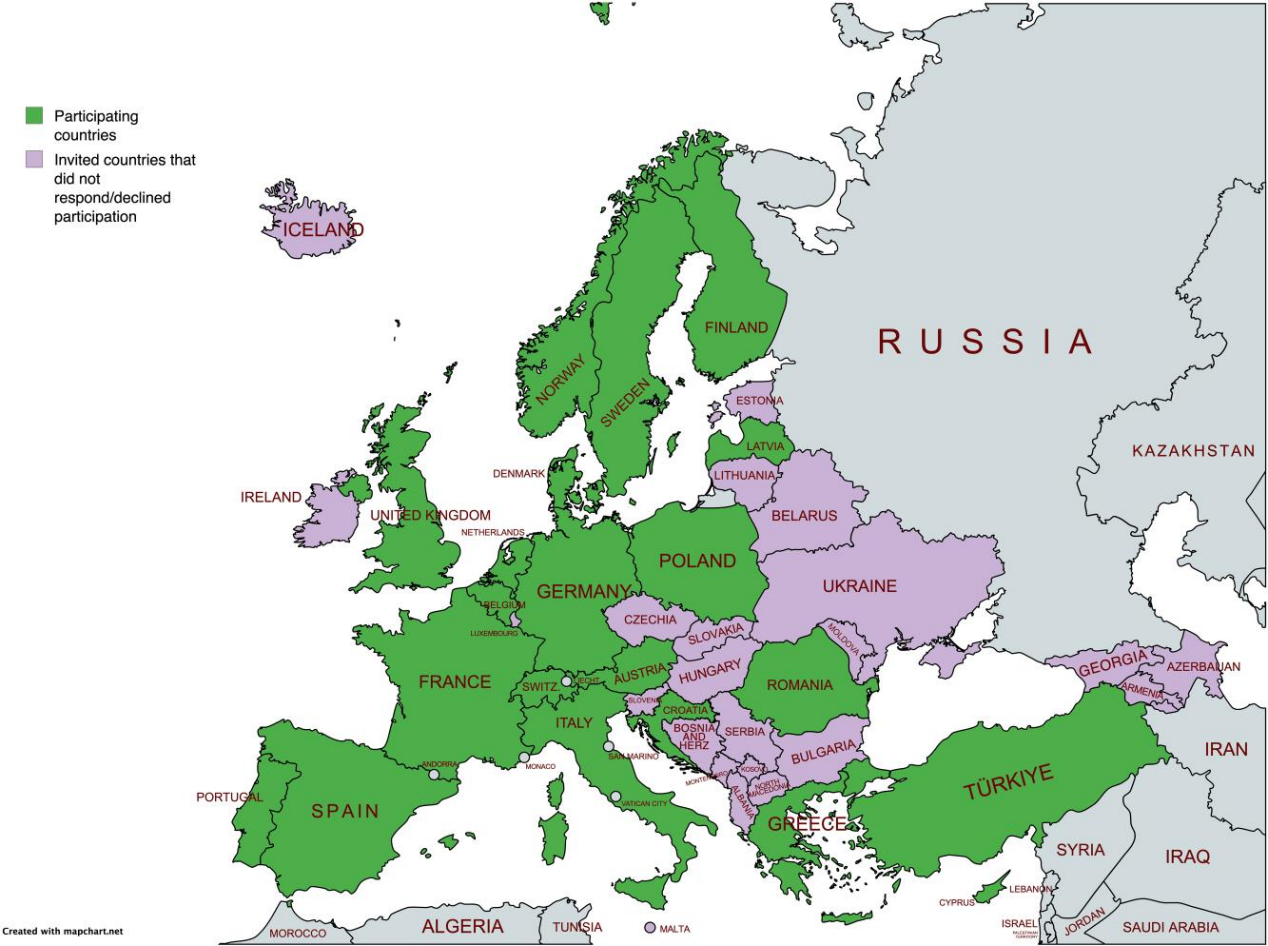
Countries that accepted and declined participation in the study LIECHT, Liechtenstein; HERZ, Herzegovina.

**Fig. 2:**
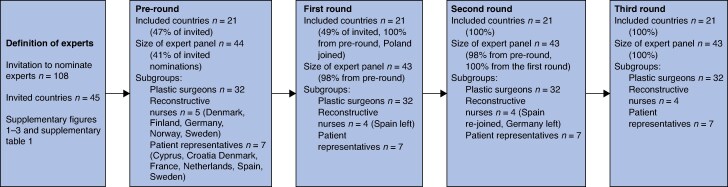
Participants in the pre-round and the three Delphi rounds

Countries recognized by the United Nations (UN) as European countries and geographical boundaries were used to define European countries. Transcontinental countries classified as European States by the UN (Azerbaijan, Georgia, and Turkey) were included. A summary of the included countries and the basis for calculating the number of nominated experts per given country is provided in *Figs S1–S3* and *Table S1*. Russia was not included as the European Commission has suspended Russian research cooperation, and the European Union (EU) has not launched any new research projects with Russian universities and researchers^16^. In addition, countries with < 100 000 inhabitants (San Marino, Andorra, Monaco, Vatican City, and Liechtenstein) were excluded. As a foundation, one expert from each included country was nominated. Additional experts were nominated based on EU membership, number of inhabitants, and healthcare expenditure. The total number of experts nominated per country is provided in *Fig. S3* and *Table S1*. The included countries are summarised in *Fig. 1* and participants in *Fig. 2*.

### Nomination process

The elected national delegates of ESPRAS, the national plastic surgical societies, and the official national patient organizations were asked to nominate experts fulfilling the criteria. There is no official organ specifically for reconstructive nurses, so these were nominated by the surgeons. All nominated surgeons were sent study information about the study and the Delphi process and formally asked whether they were willing to participate in the survey in a letter signed by the steering committee leader of the study (A. E.) and the then president of ESPRAS (R. G.). The participants are summarized in *Fig. 2*.

### Delphi technique

#### Delphi methodology

A classic Delphi methodology was used^17^. The Delphi technique is a well established scientific method for developing quality indicators used since the 1970s^6^. It involves a structured group consensus process in which a panel of experts participates in several rounds of anonymous questionnaires. Feedback is provided between rounds, and the agreement level among participants is assessed. The anonymity of the experts is a core strength of the method, reducing the influence of dominant individuals and allowing for unbiased discussion. In addition, because experts do not interact directly, the Delphi technique enables the inclusion of experts from diverse geographic regions.

The specific guidelines on using the Delphi technique to develop quality indicators suggested by Boulkedid *et al*.^6^ were followed, except that a 1–5 Likert scale was used for rating instead of the suggested 1–9 scale and questionnaires were only sent electronically and not both electronically and by regular mail. The scale was changed because new evidence regarding Delphi scales has emerged (for example Lange *et al*.^18^) since the guideline was published.

**Fig. 3:**
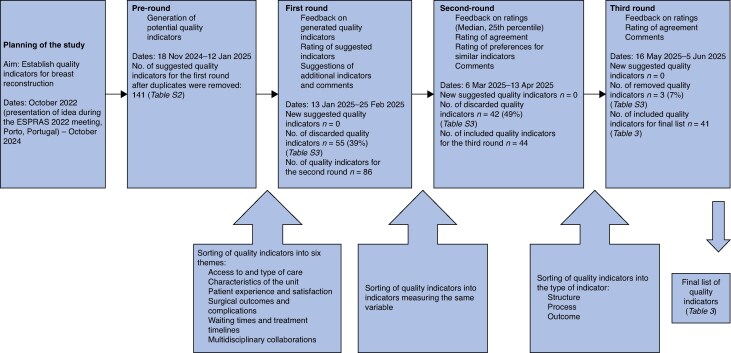
The Delphi process

It was decided that the Delphi process would include a pre-round and three subsequent rounds *a priori.* The most common methods for eliciting questionnaires for the Delphi process are to conduct a literature review on the topic or interview a small number of experts. Because there is very little published on quality indicators in breast reconstruction, it was decided not to conduct a literature review. Europe is a very diverse region, which could influence the results if interviews were performed with a limited number of experts; therefore, it was decided to elicit the Delphi questionnaire by using a modified ExpertLens technique^19^, where all the experts participated in eliciting items. The same experts participated in the pre-round and all the Delphi rounds. The Delphi rounds are summarized in *Fig. 3*. No consulting regarding the method took place.

All questionnaires were sent and answers were collected via Google Forms (Google LLC, Mountain View, CA, USA). Four reminders were sent to each expert if they did not answer the questionnaire. No physical or virtual meetings were held so that experts remained anonymous from each other and to avoid group dynamics influencing the process. No financial compensation was given to the experts. Response rates were recorded for each Delphi round. All experts who were nominated and met the definition of expert were invited to the first round. All experts who completed the previous rounds were invited to participate in the subsequent round. In the last round, all experts who participated were offered inclusion in the acknowledgement section of the manuscript if they wished, and thereby have their identity as experts revealed.

#### Definition of consensus and statistical analysis

Consensus for the different rounds was defined *a priori* and is given in *Table 2*. Because a Likert scale was used, median and interquartile ranges suitable for ordinal data were calculated. Participants graded the quality indicators on a 5-point Likert scale (from 1 (‘not important) to 5 (very important)). Missing values were given a score of 1 (not important), assuming that the expert would have actively scored it if they had thought it was important. Considerations regarding differences in views in different subgroups were incorporated in round 1 because items that did not reach consensus in the group as a whole were brought forward to the next round if a consensus was reached in at least two of the subgroups.

**Table 2:** *A priori* definitions of consensus during different rounds

	Definition of consensus	Fate of items not reaching consensus
Round 1	75%: The consensus was reached if at least 75% of participants in the total group or at least two subgroups rated the indicator as 4 or 5 (that is, when the median was 4 or 5 with a 25th percentile of ≥ 4 in the total group or in at least two subgroups)	Rejected and not included in the next round Suggested indicators with a median value of ≤ 3 and indicators with a median of 4 or 5 and a 25th percentile of < 4 were rejected^20^
Round 2	70%: The consensus level was reached if 70% of the experts preferred one of the options or agreed to the suggested quality indicator	Rejected and not included in the next round An exception was made to the preference questions; if no alternative reached consensus, several options were brought to the next round
Round 3	75%	Rejected and not included in the final list

#### Delphi questionnaire preparation: pre-round

Before the first Delphi round, the expert panel was asked to generate the indicator selection to be included in the first questionnaire. The experts were sent a questionnaire with a few examples of possible quality indicators and asked to add all other relevant quality indicators they could think of. The quality indicators were descriptively analysed by two authors (E. H. and A. E.) using thematic analysis described by Braun and Clark^21^. A deductive approach was used because it was expected that themes usually found in quality indicators would be found. The purpose of the thematization was to facilitate the presentation of the indicators to the experts in the following rounds. The wording of the experts was used with minor editing. No items were omitted except duplicates.

#### Delphi round 1

All the suggested quality indicators given in the Delphi questionnaire preparation round were sorted according to themes generated during the pre-round (qualitative feedback to the experts of suggested indicators) and included in the questionnaire. Google Forms shuffled the question order within each theme. The experts were asked to grade the importance of each suggested quality indicator on a 5-point Likert scale (from 1 (‘not important) to 5 (very important)). There was an option to comment in free-text boxes after each theme.

#### Modifications during the Delphi process

In the initial protocol, the second round was intended for experts to indicate their agreement or disagreement with the quality indicators identified in round 1. In the third round, the experts would evaluate the proposed standards of care for the indicators from round 2 and assess whether each indicator was feasible or not feasible in their respective countries. However, due to the many diverse indicators suggested during the pre-round and considered important in round 1, the process was adjusted as detailed below.

In round 2, the experts were asked to agree/disagree with the suggested quality indicators and state preferences for specific wording of similar quality indicators (for example, ‘the proportion of patients offered immediate breast reconstruction’ versus ‘the proportion of women having immediate breast reconstruction’).

The indicators were divided into structure, process, and outcome indicators^4^. Patient-reported experience measures (PREMs) were considered process indicators, and patient-reported outcome measures (PROMs) were considered outcome indicators. In round 3, the experts were asked to agree or disagree with the final quality indicators.

#### Experts’ comments during the rounds

Experts’ comments during the rounds were incorporated if they added something that had not been mentioned by any of the experts before. At the end of Delphi round 3, a thematization of the comments was performed to create background material that is easy to follow for the interested reader. This thematization was for presentation purposes only and was not used in the Delphi process.

#### Feedback to the experts during the different rounds

In round 1, all suggested indicators in the pre-round were presented to the experts. In round 2, the experts were informed about the median scores, 25th percentile, and frequencies for all the quality indicators presented in round 1 (quantitative feedback). The scores were aggregated across all expert groups and separately for the subgroups: plastic surgeons, reconstructive nurses, and patient representatives. The scores were sent to the experts in a PDF file and were presented for the entire group and subgroups as in *Tables S2* and *S3*. In round 3, the experts were informed about the agreement of preferences for the quality indicators presented in round 2, as presented in *Tables S2* and *S3* and in *Table 3*. Any new quality indicators suggested in the free-text boxes and comments were presented and included in the next round (qualitative feedback). Between the rounds, all experts received all free-text comments made during the previous round in a list in a PDF file (qualitative feedback). Information in comments that could reveal the identity of the expert was censored. The experts were not provided with information regarding which comments were made by whom.

**Table 3:** Final list of quality indicators, their scores, and agreement levels in the Delphi rounds

	Round 1 (*n* = 43)		Agreement	
	Median	25th percentile	Round 2 (*n* = 43)	Round 3 (*n* = 43)
**Structural quality indicators**				
No. of experienced surgeons in the unit	5	4	72%*	86%
No. of immediate and delayed breast reconstructions performed per year	5	4	86%	95%
No. of breast cancer cases performed per year in the unit	5	4	93%	86%
Proportion of units offering both implant-based and autologous breast reconstruction	5	4	84%	84%
System for monitoring outcomes in the unit	5	4	93%	91%
Active research in the unit	4	4	77%	86%
**Process quality indicators**				
Proportion of patients offered immediate breast reconstruction	5	4	84%	88%
Proportion of patients with access to both immediate and delayed breast reconstruction	4	4.25	84%	88%
Proportion of patients offered both immediate and delayed breast reconstruction	5	4	86%	81%
Proportion of patients with access to both implant-based and autologous breast reconstruction	5	4	86%	91%
Proportion of patients with access to genetic testing and counselling	5	5	79%	83%
Proportion of patients with access to contralateral symmetrization	5	4	71%*	91%
Proportion of patients in whom individualized treatment planning is performed	5	4	74%*	91%
Proportion of patients who receive preoperative counselling and support	4	3	84%	98%
Proportion of patients with the possibility of actively taking part in decision-making concerning breast reconstruction	5	4	81%	88%
Proportion of patients who receive information about how to prevent postoperative complications (for example, wound care, use of compression garments, mobilization)	5	4	92%	84%
Proportion of patients who have access to postoperative physiotherapy focused on breast reconstruction patients	4	3	81%	77%
Whether equal access to breast reconstruction is offered in the country	5	4	88%	79%
Waiting time from initial biopsy to surgery with immediate breast reconstruction	5	4	79%	86%
Proportion of patients who are satisfied with the healthcare staff's clarity, empathy, and support in relation to decision-making about the type of reconstruction (PREM)	5	4	82%	79%
Proportion of patients who experience adequate postoperative pain management (PREM)	4	4	84%	91%
Proportion of patients who are aware of the risks and benefits of different reconstructive options (PREM)	5	4	88%	86%
Proportion of patients who feel their own choice was respected and central to decision-making (PREM)	5	4	81%	84%
**Outcome quality indicators**				
Psychosocial impact of the reconstruction over time	4.5	4	77%	91%
Proportion of patients who would have made the same choice if asked 1–3 years later (decisional regret) (PROM)	4	3.25	71%*	83%
Proportion of patients who have restored their lifestyle after mastectomy and reconstruction regarding resumption of work, social life, sports activities (PROM)	5	4	93%	93%
Proportion of patients who are satisfied with the reconstruction (PROM)	5	4.25	98%	95%
Proportion of patients who are satisfied with the symmetry (PROM)	5	4	81%	93%
Proportion of patients who are satisfied with the aesthetic outcomes (PROM)	5	4	76%	95%
Proportion of patients who are satisfied with freedom of movement and comfort in the reconstructed area (PROM)	4	4	73%	88%
Frequency of complications according to Clavien–Dindo	4	4	91%	79%
Frequency of complications in the first 30 days	5	4	95%	86%
Frequency of complications after 30 days	4	4	83%	79%
Frequency of irradiated reconstruction (radiation after reconstruction)	4	4	77%	76%
Frequency of readmissions	4	4	77%	76%
Frequency of reoperations	5	4	79%	86%
Frequency of loss of free flaps	5	4	72%	91%
Frequency of skin necrosis in mastectomy flaps	5	4	70%*	86%
Frequency of implant loss	5	4	77%	91%
Frequency of capsular contracture	4	4	72%*	79%
Frequency of infection	5	4	74%*	93%

In round 1, participants graded the quality indicators on a five-point Likert scale (1, not important; 5, very important). In rounds 2 and 3, the participants were asked to agree or disagree with the indicator. *Quality indicators that almost reached consensus (70.0–74.9%) in round 2 and were given a second chance in round 3. PREM, patient-reported experience; PROM, patient-reported outcome.

### Evidence grading of the suggested indicators

Evidence grading of the suggested indicators will be performed in a separate process and published in a separate paper.

## Results

Forty-five European countries were invited and 21 (47%) agreed to participate (*Fig. 1*). Among EU countries and European Economic Area (EEA)/single-market countries, 63% (19 of 30) participated, whereas among non-EU/non-EEA/non-single-market European countries, 13% (2 of 15) participated. In all, 43 experts answered all the questionnaires, of whom 7 were patient representatives, 4–5 (depending on round) were reconstructive nurses, and the rest were plastic surgeons (*Fig. 2*).

In the pre-round, 141 unique quality indicators were suggested. Six themes could be identified: Access to and type of care (50 quality indicators); Waiting times and treatment timelines (11); Multidisciplinary collaborations (6); Characteristics of the unit (12); Surgical outcomes and complications (37); and Patient experience and satisfaction (25) (*Fig. 3* and *Table S2*). In Delphi round 1, the consensus (75%) was reached for 86 quality indicators, with a median of 4 or 5 and a 25th percentile of ≥ 4 in the total group (74 indicators) or at least two subgroups (12 indicators) (*Fig. 3* and *Table S2*). In Delphi round 2, the consensus (75%) was reached for 34 indicators. In addition, indicators almost reaching consensus (70–74.9%) were included, resulting in 44 indicators for round 3. In Delphi round 3, the consensus (75%) was reached for 41 indicators (*Table 3*). Discarded quality indicators are provided in *Table S3*. A qualitative analysis of the experts’ comments is provided in the *supplementary results*.

## Discussion

This study developed a comprehensive set of 41 quality indicators for breast reconstruction through a structured Delphi consensus process involving a diverse panel of European experts, including surgeons, nurses, and patients. This is the first initiative to address the substantial gap in validated quality indicators for breast reconstruction care in Europe. The resulting indicators encompass structure, process, and outcome domains as outlined in Donabedian’s model, offering a multidimensional framework for evaluating and improving breast reconstruction services. Further research is needed to turn the suggested quality indicators into a practical, usable set of indicators that can be implemented.

The diversity and breadth of the final 41 indicators underscore the complexity of breast reconstruction and the need for a nuanced approach to quality assessment. Previously suggested indicators for breast reconstruction^9^ focus narrowly on procedure rates, which risks overlooking other critical dimensions such as patient preferences, complication rates, access disparities, and long-term outcomes. In contrast, the indicators identified in the present study reflect a broader and more patient-centred conceptualization of quality, including indicators for PREMs and PROMS (patient-centredness), as well as the other domains of quality in healthcare: safety, timeliness, effectiveness, efficiency, and equity^2^. Similarly, the Organization for Economic Co-operation and Development (OECD) supports integrating patient-reported measures into system performance assessments^22^. By including both technical outcomes (for example, complication rates and revision surgeries) and patient-centred metrics (for example, satisfaction and involvement in decision-making), the final set provides a balanced view that may help mitigate risks of measurement fixation^8^ and a tendency to lean towards certain types of care for the sake of performance.

In relation to the BRAVO core outcome set for research and audit studies in breast reconstruction^14^, the suggested quality indicators cover all the core items important for both patients and professionals (major complications, unplanned surgery for any reason, donor site problems/morbidity, normality, quality of life, and women’s cosmetic satisfaction), for professionals only (implant-related complications and flap-related complications), and for patients only (self-esteem, emotional wellbeing, and physical wellbeing)^14^. Similar to the BRAVO study, the present study does not consider when and how the outcomes/quality indicators should be measured. Moreover, the indicators align with the ICHOM^13^ and OECD^22^ recommendations, as well as with the previously suggested quality indicators specifically for autologous breast reconstruction developed in a Swiss study^23^.

In this paper the quality indicators are presented as the experts recommended them, and no consideration has yet been given to how the outcomes should be measured. As a result, some of the quality indicators somewhat overlap. A few quality indicators may be suited to becoming composite indicators^3^. For example, some patient satisfaction indicators, such as the proportions of patients satisfied with the reconstruction, symmetry, aesthetic outcome, freedom of movement, and comfort, may be measured with an instrument capturing all these aspects. Similarly, some of the complication indicators overlap. For example, complications, according to the Clavien–Dindo classification system, include the frequency of complications within the first 30 days and the frequency of reoperations^24^. Further research is needed regarding which aspects warrant separate reporting and which indicators could be suitable to becoming composite indicators.

A significant strength of this study lies in the rigorous application of the Delphi technique, following established methodological recommendations^6^ and reporting guidelines (DELPHISTAR)^15^. Including diverse stakeholders (plastic surgeons mastering all types of breast reconstruction, reconstructive nurses, and patient representatives) from across 21 European countries ensured that various perspectives were captured, enhancing both the content validity and the acceptability of the indicators. Using anonymized, multiround feedback mitigated the risk of dominance by specific individuals or national practices and promoted unbiased consensus formation. In addition, using quantitative and qualitative feedback across Delphi rounds facilitated the refinement of indicators while maintaining transparency in decision-making. This iterative process enabled the retention of high-priority indicators and excluded those lacking sufficient support, ensuring that the final indicators are relevant in diverse healthcare settings.

Despite the study’s strengths, several limitations should be acknowledged. First, although the Delphi technique offers a robust method for achieving expert consensus, it does not substitute for empirical validation. The indicators identified in this study will require further testing for reliability, validity, feasibility, and responsiveness in clinical practice. Plans for evidence grading and validation in subsequent phases are therefore essential. Second, the scope of the study was to create European quality indicators. Europe is a widely diverse region with varying healthcare infrastructure, reimbursement models, cultural attitudes towards breast reconstruction, and different specialities performing breast reconstruction, making it challenging to create indicators usable for the entire area. Nonetheless, a well over 80% consensus was reached for most of the indicators included, suggesting that they are useful in most European countries. Similarly, although a structured and criteria-based approach to expert selection and the number of invited countries were used, some countries and professional subgroups were under-represented, which may influence the applicability of specific indicators. In the study, the medical experts were defined as board-certified plastic surgeons who performed all types of breast reconstruction. It could be argued that other specialities that perform implant-based breast reconstruction should also be included. However, it was decided to include experts who are trained as subspecialist plastic surgeons who perform all types of reconstructions in this study to avoid the possibility that an expert’s skill in performing only one type of reconstruction influences the results. Moreover, the limited number of experts (34) could have limited the generalizability of the results. Initially, a greater number of experts was planned, and considerable efforts were made to recruit them. To enable the inclusion of more experts, the transparent and official channels to include them would have had to have been abandoned, and the stringent requirements regarding the definition of an expert (for example, more than 5 years as a subspecialist plastic surgeon) would have had to have been relaxed. The steering committee made the decision to keep the strict recruitment channels and requirements regarding experts, even if it was at the price of not being able to recruit as many experts as initially planned. Third, the study did not consider the feasibility of measuring the indicators. For example, although PROMs and PREMs were incorporated as core components, the feasibility of collecting these systematically is challenging in most European healthcare systems. Future implementation efforts should consider the infrastructure needed to gather, interpret, and act on patient-reported data, as well as the general feasibility of measuring the indicators.

**Fig. 4:**
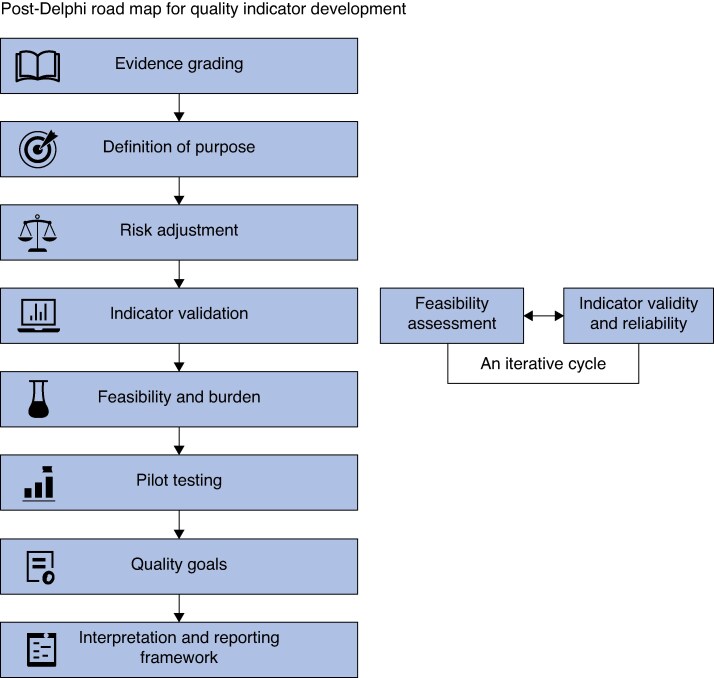
Methodological and practical considerations to address before implementation

Several methodological and practical considerations must be addressed before the quality indicators can be implemented in clinical practice. These include evidence grading, definition of purpose, risk adjustment, validation, data collection feasibility, and the development of quality benchmarks and interpretation frameworks (*Fig. 4*). For quality indicators to be meaningful and credible, they must be underpinned by systematically evaluated evidence. This includes linking each indicator to relevant scientific literature or clinical guidelines. A structured evidence grading system, such as GRADE^25^, needs to be applied. The evidence grading forms a basis for the minimum and target levels that can be set for the indicators. For example, to set a high minimum level for an indicator, it needs to be supported by strong scientific evidence. Moreover, the final indicators should be explicitly categorized according to their intended use: external quality assurance (for example, benchmarking across institutions, national audits) and/or for internal quality improvement (for example, local monitoring and learning)^3^. Given the variability in patient populations and clinical presentations, risk adjustment is essential for fair and relevant comparison across healthcare providers and/or systems^26^. Particularly in breast reconstruction, patient-level variables such as age, body mass index, smoking status, co-morbidities, cancer staging, and treatment history (for example, radiation therapy) significantly impact outcomes. Indicators must also be assessed for validity (that is, they measure what they are intended to measure) and reliability (that is, they yield consistent results across different settings and times)^27,28^. Content validity was addressed through the multistakeholder Delphi consensus process. However, further work is required to evaluate validity and reliability through empirical studies. Part of the validation process includes defining how each indicator should be measured, such as how different complications should be defined^29^. A significant determinant of whether an indicator is actionable is its feasibility. Structured feasibility assessments using established instruments^30^ can help evaluate aspects such as data availability in electronic health records, the need for manual abstraction, the cost of data collection, and the clarity of definitions. Before widespread adoption, selected indicators should undergo pilot testing in clinical settings^30^. Defining minimum and target levels is necessary to guide performance improvement. These thresholds should be evidence informed and developed using historical data, expert consensus, and input from stakeholders. Finally, a standardized appraisal and reporting framework is needed to enable the meaningful use of indicators^3^. In the longer term, the indicators provide a foundation for creating standardized audits, benchmarking efforts, and quality improvement initiatives in breast reconstruction.

## Collaborators

The members of the ESPRAS European Quality Indicator group are: Tanja Aaby Orellana (Department of Plastic Surgery, Sahlgrenska University Hospital, Gothenburg, Sweden); João Correia Anacleto (Champalimaud Foundation, Hospital Cuf Cascais, Portugal); Andreas Arkudas (Department of Plastic and Hand Surgery, University Hospital of Erlangen, Friedrich-Alexander-University Erlangen-Nürnberg, Erlangen, Germany); Giovanni Di Benedetto (Univesità Politecnica delle Marche, Ancona, Italy); Couturaud Benoit (Curie Insititut, Paris, France); Linda Bull Madsen (Department of Plastic Surgery, Haukeland University Hospital, Bergen, Norway); Burcu Celet Ozden (Istanbul Breast Clinic and VKV American Hospital Breast Unit, Istanbul, Türkiye); Giovanni Di Benedetto (Department of Experimental and Clinical Medicine, Clinic of Plastic and Reconstructive Surgery, Marche Polytechnic University Medical School, Ancona, Italy); Ann-Charlott Docherty Skogh (Department of Reconstructive Plastic Surgery and Maxillofacial Surgery, Karolinska University Hospital and Karolinska Institutet, Stockholm, Sweden); Tine Engberg Damsgaard (Department of Plastic Surgery, Odense University Hospital, Odense; University Hospital of Southern Denmark, SLBVejle, Sygehus Lillebaelt Vejle; and Department of Regional Health Research, University of Southern Denmark, Odense, Denmark); Patricia García Marcos (General University Hospital Gregorio Marañón, Madrid, Spain); Ansis Gilis, Pauls Stradiņš Clinical University Hospital, Riga, Latvia); Adam Gilmour (Canniesburn Plastic Surgery Unit, North Glasgow University Hospitals, Glasgow, UK); C. Guerrero Paez (Dutch Breast Cancer Society, Utrecht, Netherlands); Moustapha Hamdi (University Hospital Brussels, KidZ Health Castle Children's Hospital, Free University Brussels, Brussels Health Campus, Brussels, Belgium); Yves Harder (Department of Plastic, Reconstructive and Aesthetic Surgery and Hand Surgery, Centre Hospitalier Universitaire Vaudois, Lausanne, Switzerland); Cristian Radu Jecan (Carol Davila University of Medicine, Bucharest, Romania); Ulla Karhunen-Enckell (Breast Surgery Department, Tampere University Hospital, University of Tampere, Tampere, Finland); Christian Korvald (Department of Plastic and Reconstructive Surgery, Oslo University Hospital, Oslo, Norway); Francisco Leyva (Department of Plastic Surgery, La Paz University Hospital, Madrid, Spain); Daniel Maliszewski (Department of Surgical Oncology, Janusz Korczak Provincial Specialist Hospital, Słupsk, Poland); Maria Mani (Department of Plastic and Reconstructive Surgery, Uppsala University and Uppsala University Hospital, Uppsala, Sweden); Päivi Merkkola-von Schantz (Department of Plastic and Reconstructive Surgery, Helsinki University Hospital and University of Helsinki, Helsinki, Finland); Camilla Morken Kristoffersen (Haukeland University Hospital, Bergen, Norway); Xavier Nelissen (CHU Liège, Liège, Belgium); Laura Pandis (Plastic and Reconstructive Surgery, University of Padua, Padova, Italy); Nikolaos A. Papadopulos (Department of Plastic Surgery, Attikon and Eugenideio University Hospitals, School of Medicine, National and Kapodistrian University of Athens, Athens, Greece); Sari Pieper (Department of Breast Surgery, Tampere University Hospital, Tampere, Finland); Hinne Rakhorst (UMCG Groningen, UMCG Groningen, Netherlands); Maurizio Ressa (I.R.C.C.S. Istituto Tumori ‘Giovanni Paolo II’, Bari, Italy); Marzia Salgarello (Catholic University of the Sacred Heart, Plastic Surgery Department, University Hospital A. Gemelli, Rome, Italy); Fabio Santanelli di Pompeo (‘Sapienza’ University of Rome, Rome, Italy); Kavita Sharma (Canniesburn Plastic Surgery Unit, North Glasgow University Hospitals, Glasgow, UK); Thomas Sjøberg (Department of Plastic and Reconstructive Surgery, University Hospital of North Norway, Tromsø, Norway); Karen Sundbøll (Danish Breast Cancer Association, Trekantområdet, Denmark); Cristina Triviño de la Cal (Spanish Breast Cancer Association, Santander, Spain); Maja Erceg Tušek, Centre for Psychological Assistance, Association of Cancer Affected and Treated Women EVERYTHING for HER, Zagreb, Croatia; Ruth Waters (BAPRAS, The University Hospital Birmingham, Birmingham, UK); Carlos Villar Yus (Hospital Universitario La Paz, Madrid, Spain); Dolores Wolfram (Department of Plastic, Reconstructive and Aesthetic Surgery, Medical University of Innsbruck, Innsbruck, Austria)

## Supplementary Material

zraf144_Supplementary_Data

## Data Availability

All data are available in the article and the supplementary files.

## References

[1] World Health Organization . Fact Sheets: Quality Health Services. https://www.who.int/news-room/fact-sheets/detail/quality-health-services (accessed 29 October 2025)

[2] Institute of Medicine (US) Committee on Quality of Health Care in America . Crossing the Quality Chasm: A New Health System for the 21st Century. Washington, DC: National Academies Press (US), 2001 25057539

[3] Organisation for Economic Co-operation and Development (OECD) and World Health Organization (WHO) . Improving Healthcare Quality in Europe: Characteristics, Effectiveness and Implementation of Different Strategies. Geneva: OECD Publishing, 2019 31721544

[4] Donabedian A . The Definition of Quality and Approaches to Its Assessment. Vol 1. Explorations in Quality Assessment and Monitoring. Ann Arbor, MI: Health Administration Press, 1980

[5] Mainz J . Defining and classifying clinical indicators for quality improvement. Int J Qual Health Care 2003;15:523–530 14660535 10.1093/intqhc/mzg081

[6] Boulkedid R, Abdoul H, Loustau M, Sibony O, Alberti C. Using and reporting the Delphi method for selecting healthcare quality indicators: a systematic review. PLoS One 2011;6:e20476 21694759 10.1371/journal.pone.0020476 PMC3111406

[7] Woolf S, Schunemann HJ, Eccles MP, Grimshaw JM, Shekelle P. Developing clinical practice guidelines: types of evidence and outcomes; values and economics, synthesis, grading, and presentation and deriving recommendations. Implement Sci 2012;7:61 22762158 10.1186/1748-5908-7-61 PMC3436711

[8] Mannion R, Braithwaite J. Unintended consequences of performance measurement in healthcare: 20 salutary lessons from the English National Health Service. Intern Med J 2012;42:569–574 22616961 10.1111/j.1445-5994.2012.02766.x

[9] Rubio IT, Marotti L, Biganzoli L, Aristei C, Athanasiou A, Campbell C et al EUSOMA quality indicators for non-metastatic breast cancer: an update. Eur J Cancer 2024;198:113500 38199146 10.1016/j.ejca.2023.113500

[10] Mainz J . Developing evidence-based clinical indicators: a state of the art methods primer. Int J Qual Health Care 2003;15 (Suppl 1):i5–11 14660518 10.1093/intqhc/mzg084

[11] Biganzoli L, Marotti L, Hart CD, Cataliotti L, Cutuli B, Kuhn T et al Quality indicators in breast cancer care: an update from the EUSOMA working group. Eur J Cancer 2017;86:59–81 28963914 10.1016/j.ejca.2017.08.017

[12] DeKoning J, Burgers J, Klazinga N. Development and validation of a measurement instrument for appraising indicator quality: appraisal of indicators through research and evaluation (AIRE) instrument. In: *Proceedings of the* Kongress Medizin und Gesellschaft, Augsburg, 2007.

[13] Ong WL, Schouwenburg MG, van Bommel ACM, Stowell C, Allison KH, Benn KE et al A standard set of value-based patient-centered outcomes for breast cancer: the International Consortium for Health Outcomes Measurement (ICHOM) initiative. JAMA Oncol 2017;3:677–685 28033439 10.1001/jamaoncol.2016.4851

[14] Potter S, Holcombe C, Ward JA, Blazeby JM; BRAVO Steering Group. Development of a core outcome set for research and audit studies in reconstructive breast surgery. Br J Surg 2015;102:1360–1371 26179938 10.1002/bjs.9883 PMC5034747

[15] Niederberger M, Schifano J, Deckert S, Hirt J, Homberg A, Koberich S et al Delphi studies in social and health sciences—recommendations for an interdisciplinary standardized reporting (DELPHISTAR). Results of a Delphi study. PLoS One 2024;19:e0304651 10.1371/journal.pone.0304651 PMC11346927 39186713

[16] European Commission . *Russia*. https://research-and-innovation.ec.europa.eu/strategy/strategy-research-and-innovation/europe-world/international-cooperation/bilateral-cooperation-science-and-technology-agreements-non-eu-countries/russia_en (accessed 29 October 2025)

[17] Dalkey N, Helmer O. An experimental application of the Delphi method to the use of experts. Manage Sci 1963;9:458–467

[18] Lange T, Kopkow C, Lutzner J, Gunther KP, Gravius S, Scharf HP et al Comparison of different rating scales for the use in Delphi studies: different scales lead to different consensus and show different test-retest reliability. BMC Med Res Methodol 2020;20:28 32041541 10.1186/s12874-020-0912-8 PMC7011537

[19] Dalal S, Khodyakov D, Srinivasan R, Straus S, Adams J. ExpertLens: a system for eliciting opinions from a large pool of non-collocated experts with diverse knowledge. Technol Forecast Soc Change 2011;78:1426–1444

[20] Lavan AH, Gallagher P, Parsons C, O'Mahony D. STOPPFrail (Screening Tool of Older Persons Prescriptions in Frail adults with limited life expectancy): consensus validation. Age Ageing 2017;46:600–607 28119312 10.1093/ageing/afx005

[21] Braun V, Clarke V. Using thematic analysis in psychology. Qual Res Psychol 2006;3:77–101

[22] Organisation for Economic Co-operation and Development (OECD) . Measuring What Matters: The Patient-Reported Indicator Surveys. Patient-Reported Indicators for Assessing Health System Performance. Geneva: OECD Publishing, 2019

[23] Lindenblatt N, Leuenberger NJ, Harder Y, Kappos EA, Pusic AL, Shaw J et al Measuring quality of care in autologous breast reconstruction: a Delphi consensus. Eur J Surg Oncol 2024;50:107254 38056020 10.1016/j.ejso.2023.107254

[24] Clavien PA, Barkun J, de Oliveira ML, Vauthey JN, Dindo D, Schulick RD et al The Clavien–Dindo classification of surgical complications: five-year experience. Ann Surg 2009;250:187–196 19638912 10.1097/SLA.0b013e3181b13ca2

[25] Guyatt GH, Oxman AD, Vist GE, Kunz R, Falck-Ytter Y, Alonso-Coello P et al GRADE: an emerging consensus on rating quality of evidence and strength of recommendations. BMJ 2008;336:924–926 18436948 10.1136/bmj.39489.470347.AD PMC2335261

[26] Lezzoni LI . Risk Adjustment for Measuring Health Care Outcomes. Arlington, VA: Health Administration Press (Foundation of the American College of Healthcare Executives (ACHE), 2003

[27] Agency for Healthcare Research and Quality (AHRQ) . *AHRQ Quality Indicators Toolkit for Hospitals.* https://www.ahrq.gov/patient-safety/settings/hospital/resource/qitool/index.html (accessed 29 October 2025)

[28] Organisation for Economic Co-operation and Development (OECD) . Handbook on Constructing Composite Indicators: Methodology and User Guide. Geneva: OECD Publishing, 2008

[29] Potter S, Brigic A, Whiting PF, Cawthorn SJ, Avery KN, Donovan JL et al Reporting clinical outcomes of breast reconstruction: a systematic review. J Natl Cancer Inst 2011;103:31–46 21131574 10.1093/jnci/djq438

[30] Campbell SM, Kontopantelis E, Hannon K, Burke M, Barber A, Lester HE. Framework and indicator testing protocol for developing and piloting quality indicators for the UK quality and outcomes framework. BMC Fam Pract 2011;12:85 21831317 10.1186/1471-2296-12-85 PMC3176158

