# Supplementary material for: Quality indicators for breast reconstruction following cancer—an international Delphi consensus study supported by the European Society of Plastic, Reconstructive and Aesthetic Surgery
Source: BJS Open. 2025 Nov 24;9(6):zraf144. doi: 10.1093/bjsopen/zraf144 (PMC12641120; doi:10.1093/bjsopen/zraf144)
Supplement: zraf144_Supplementary_Data [file zraf144_supplementary_data.docx]

**Quality indicators for breast reconstruction following cancer - an international Delphi consensus study supported by the European Society of Plastic, Reconstructive and Aesthetic Surgery (ESPRAS)**

Emma Hansson^1,2^, MD, PhD, Nicholas Moellhoff^3^, MD, PhD, Susanne Ahlstedt Karlsson^1,2,4^, RN, PhD, Alexandra Uusimäki^5^, Ilkka Kaartinen^6^, MD, PhD, Lisbet Rosenkrantz Hölmich^7^, MD, PhD, The ESPRAS European Quality Indicator Group (ESPRAS-EQIG)^*^, Rado Zic^8^, MD, PhD, Ruth Waters^9^, MD, Mark Henley^10^, MD, Riccardo E. Giunta^3^, MD, PhD, Anna Elander^1,2^, MD, PhD

^1. Department of Plastic Surgery, Institute of Clinical Sciences, The Sahlgrenska Academy, University of Gothenburg, Gothenburg, Sweden^

^2. Department of Plastic Surgery, Sahlgrenska University Hospital, Gothenburg, Region Västra Götaland, Sweden^

^3. Division of Hand, Plastic and Aesthetic Surgery, University Hospital, LMU Munich, Germany.^

^4. Institute of Health and Care Sciences, Sahlgrenska Academy, University of Gothenburg, Gothenburg, Sweden^

^5. Johanna, Gothenburg branch of the Swedish Breast Cancer Association^

^6. Department of Musculoskeletal Surgery and Diseases, Clinic of Plastic and Reconstructive Surgery, Tampere University Hospital, Pirkanmaa Hospital District, Tampere, Finland.^

^7. Department of Plastic Surgery, Copenhagen University Hospital, Herlev, Denmark.^

^8. Croatia University Hospital Dubrava, Department of Plastic, Reconstructive, and Aesthetic Surgery, Croatia^

^9. Queen Elizabeth Hospital, University Hospitals Birmingham NHS Foundation Trust, Birmingham, UK^

^10. Department of Plastic and Reconstructive Surgery, Nottingham University Hospitals NHS Trust, Nottingham, UK^

^* Members of the ESPRAS European Quality Indicator Group (ESPRAS-EQIG) are co-authors of this study and can be found under Collaborators^

**Corresponding author.** Emma Hansson, Department of Plastic Surgery, Sahlgrenska University Hospital, Röda Stråket 12, SE-413 45 Gothenburg, Sweden. Tel: +46 31 342 10 00 Fax: +46 31 82 79 03

E-mail: emma.hansson.2@gu.se

**ORCID ID 0000-0002-3218-0881**

**Supplementary Materials – Index**

[Supplementary results 2](#_Toc210673895)

[Supplementary results 1 : Qualitative analysis of the experts’ comments according to Braun and Clark 2](#_Toc210673896)

[Supplementary Figures and Tables 9](#_Toc210673919)

[Supplementary Figure 1. Nominated experts according to healthcare expenditure per capita 9](#_Toc210673920)

[Supplementary Figure 2. Nominated experts according to EU membership 10](#_Toc210673921)

[Supplementary Figure 3. Total number of nominated experts per country 10](#_Toc210673922)

[Supplementary Table 1. Nominated experts per country and type 11](#_Toc210673923)

[Supplementary Table 2: Suggested quality indicators in pre-round and scores in Delphi round 1 16](#_Toc210673924)

[Supplementary Table 3: Discarded quality indicators in the different Delphi rounds 24](#_Toc210673925)

[Supplementary Appendices 28](#_Toc210673926)

[Supplementary Appendix 1: Delphistar checklist 28](#_Toc210673927)

[Supplementary appendix 2: Steering committee 34](#_Toc210673928)

Supplementary results

## Supplementary results 1 : Qualitative analysis of the experts’ comments according to Braun and Clark

Braun V, Clarke V. Using thematic analysis in psychology. Qualitative research in psychology. 2006;3(2):77-101.

Round 1 comments

Key codes identified:

- Patient autonomy
- Multidisciplinary team involvement
- Differences across healthcare systems
- Reconstruction options and timing
- Long-term outcomes
- Communication and expectation management
- Measurement and data collection challenges
- Use of PROMs
- Complications and follow-up care

Theme 1: Informed Patient Choice and Shared Decision-Making

*Subthemes:*

- Offering all reconstruction options with full transparency
- Emphasis on **patient preferences** over provider-driven decisions
- Recognition of breast reconstruction as a **staged and long-term** process
- The need for **face-to-face education** and **peer-to-peer support**

*Illustrative quotes:*

- *"The important thing is that patients are well informed and offered all modalities of breast reconstruction..."*
- *"We should focus on whether options are offered and the choice for treatment is shown to be based on patient preference..."*
- *"Probably the best way to inform the patient... face to face, patient with patient"*

Theme 2: Access to and Type of Care Across Systems

*Subthemes:*

- Variability in access due to **health system differences**
- Call for **European data** on reconstruction timing (immediate, delayed, delayed-immediate)
- Access to **specialist expertise** (e.g., plastic surgeons)

*Illustrative quotes:*

- *"Different countries have different health systems..."*
- *"I would recommend to differentiate between immediate, delayed immediate and delayed autologous BR."*
- *"Plastic surgeons are part of the interdisciplinary Breast Center... but this is not true for most countries"*

Theme 3: Multidisciplinary Team-Based Care

*Subthemes:*

- Integration of **plastic surgeons from diagnosis to follow-up**
- Team approach including **oncology, radiology, surgery, psychology, pharmacy**
- Importance of experienced specialists for quality outcomes

*Illustrative quotes:*

- *"All involved doctors should participate... plastic surgeon, oncologist, radiotherapist..."*
- *"Best treatment is offered with a multidisciplinary team, each member of which is highly experienced..."*

Theme 4: Quality Monitoring, Complications, and Data Capture

*Subthemes:*

- Frustration with vague or hard-to-measure indicators
- Preference for standardized systems like **Clavien-Dindo**
- Focus on **impactful outcomes**, like delays to adjuvant therapy
- Acknowledgment of **minor revisions** and complication management as routine
- Questions about how to **define and measure** complications like flap necrosis or volume loss

*Illustrative quotes:*

- *"Clavien-Dindo makes sense."*
- *"Minor revisions cannot be avoided..."*
- *"The problem is, how do you diagnose the complication..."*
- *"They do not seem quality indicators for BR."*

Theme 5: Long-Term Outcomes and Patient-Reported Measures

*Subthemes:*

- Inclusion of **Patient-Reported Outcome Measures (PROMs)** like Breast-Q
- Recommendation to use **additional psychosocial tools**
- Long-term follow-up as a **standard of care**
- Managing patient expectations for **ongoing interventions**

*Illustrative quotes:*

- *"We should focus more on PROMs (!!longterm outcome)"*
- *"Reconstruction is a marathon not a sprint..."*
- *"We will be there for her in the long-term follow-up."*

Summary of Key Themes and Interpretations

| **Theme** | **Core Insight** |
| --- | --- |
| Informed Choice | Patients must receive full, individualized information and be empowered in decisions |
| Systemic Access | Care availability is uneven; European-specific data and system-level improvements are needed |
| Team-Based Care | Multidisciplinary integration is essential for safe, high-quality reconstruction |
| Measurement & Indicators | Data should be meaningful, standardizable, and focused on patient impact |
| Long-Term Support | Reconstruction success depends on prolonged care and patient-centered outcome tracking |

Round 2 comments

Codes identified:

- **Access vs. offer** – Women being offered vs. having access to reconstruction.
- **Timing** – Immediate vs. delayed reconstruction and its influencing factors.
- **Multidisciplinary care** – Importance of involving plastic surgeons early and consistently.
- **Bias in counseling** – How first contact with non-plastic surgeons affects reconstruction options.
- **Patient-centered decision-making** – Emphasis on personalized approaches, informed consent, and shared decisions.
- **PROMs and quality** – Using outcomes and satisfaction to guide future care.
- **Resources and equity** – Limitations in public healthcare systems, impact of surgeon expertise and availability.

Theme 1: **Patient-Centered Access and Equity**

- - Includes: “offered,” “access,” “bias,” “timing,” “equity,” “choice,” “adequate information,” “availability.”

**Theme 2: Multidisciplinary, Personalized Care and Communication**

- - Includes: “team involvement,” “plastic surgeon timing,” “shared decision-making,” “education,” “counseling,” “WhatsApp group,” “trust.”

**Theme 3: Outcome Quality and Monitoring**

- - Includes: “PROMs,” “return to work,” “symmetry,” “aesthetic outcome,” “post-op physiotherapy,” “photos,” “research/data feedback.”

Upon reviewing, **Theme 1** appears to be most aligned with indicators. It's clear the distinction between being *offered* and *having access to* BR is a key issue. Access includes actual capability to undergo BR, not just being informed. Offer may be dependent on surgeon bias or limited options. Theme 2 supports the first by showing how access and offering must be informed by a team approach. Theme 3 is important but more about measurement tools and not a stand-alone indicator.

Based on the analysis, the **two most important indicators** are:

**1. Proportion of Women Who Are Offered Breast Reconstruction (any type)**

- Reflects: clinical responsibility to inform, reduce bias, promote autonomy.
- Importance: If not offered, no further steps can occur. Counselling must be neutral, inclusive of all options including no reconstruction.
- Why this, not just access? Because offer is the **first gateway** to true access.

**2. Proportion of Women Who Have Access to Breast Reconstruction**

- Reflects: systemic capacity, geographic/economic availability, options (autologous vs. implant), timing (immediate/delayed).
- Importance: Having BR offered doesn’t mean it is accessible (e.g., surgeon expertise, resources, referrals).
- Includes: institutional arrangements to refer for procedures they do not offer, public system support.

These two indicators together measure **equity, quality, and autonomy**.

“Offer” is about **counseling integrity**, “Access” is about **system capability**.

Other themes (PROMs, physiotherapy, follow-up, etc.) are **supportive process indicators** but not as foundational.

Round 3 comments

Identified codes:

- Definition and monitoring of complications
- Structural integration of plastic surgery into breast cancer care
- Selection bias in reconstructive options
- Patient autonomy and informed choice
- Variability in international practice and healthcare systems
- Importance of PROMs (Patient-Reported Outcome Measures)
- Long-term vs short-term outcomes
- Economic and systemic disparities
- Role clarity and multidisciplinary coordination

**Theme 1: The Need for Clear, Standardised Definitions and Quality Indicators**

***Subthemes:***

- Importance of standardised definitions of complications
- Structural and process quality indicators for benchmarking
- Timeframes: preference for 60-day or 1-year outcomes over 30-day

***Quote:***

"Clavien Dindo classifies any re-operation... Instead of complications after 30 days, the term long-term complications/outcome (minimum 1 year postop) seems more adequate."

**Theme 2: Structural Gaps and Role of Plastic Surgeons in Breast Centers**

***Subthemes:***

- Plastic surgeons not consistently involved from diagnosis
- Gynecologists/breast surgeons acting as gatekeepers to certain procedures
- Lack of integrated consultations leads to biased offerings

***Quote:***

"The best setting is if you as a surgeon offer all reconstructive procedures or expose the patient to a common consultation with both specialists..."

**Theme 3: Patient-Centered Decision-Making and Individualized Care**

***Subthemes:***

- Respect for patient preferences and fears
- Need for breast care nurse counseling
- Surgeon bias affects options presented
- Need for shared decision-making tools

***Quote:***

"It is very important for the patients, to have the possibility to take an active part in decision-making concerning breast reconstruction."

**Theme 4: Health Equity and Access in Different Systems**

***Subthemes:***

- Reconstruction not equally accessible in all countries
- Insurance coverage disparities
- Influence of local healthcare systems and mentalities

***Quote:***

"In an ideal world, we would obviously prefer that all procedures... be in equal reach of each individual in the country."

**Theme 5: Importance of Long-Term, Multidisciplinary Outcome Monitoring**

***Subthemes:***

- Adjuvant therapies (radiation, hormonal) influence late outcomes
- Multidisciplinary responsibility for complications
- Need for long-term follow-up and accountability

***Quote:***

"Complication management... is a multidisciplinary responsibility and should be taken care of timely as a whole team."

In conclusion, this analysis illustrates a pressing need to redefine structural and process quality in breast reconstruction, moving from reactive, discipline-bound care toward an integrated, patient-centred approach. Without structural reforms, including early involvement of plastic surgeons and common consultations, many patients risk receiving incomplete or biased information about their reconstructive options. Furthermore, economic and geographic health disparities significantly influence care equity. Importantly, outcome evaluation should extend beyond short-term complications to long-term physical and psychosocial well-being, requiring robust, multidisciplinary monitoring.

# Supplementary Figures and Tables

## Supplementary Figure 1. Nominated experts according to healthcare expenditure per capita


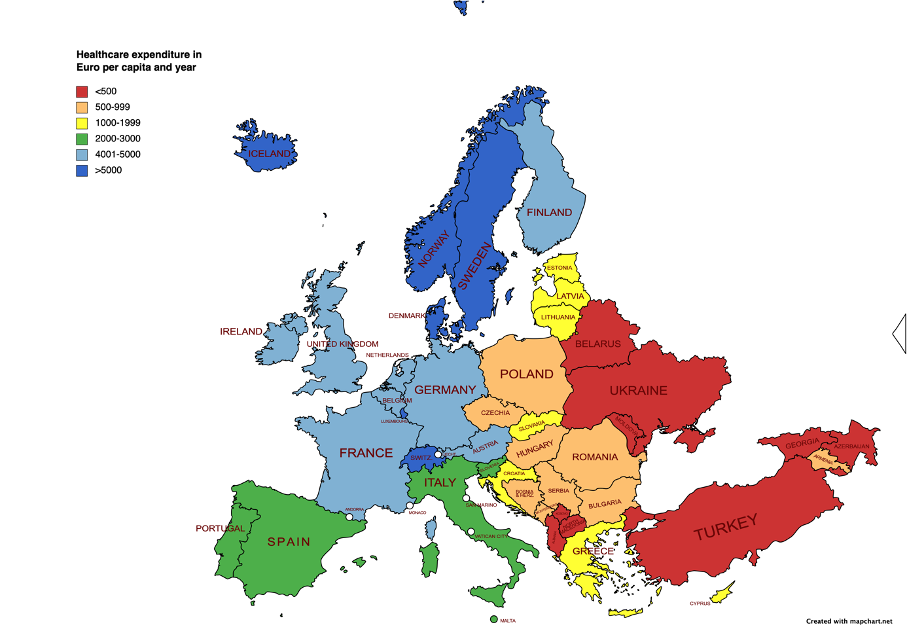


Supplementary Figure 1. Healthcare expenditure in Euro per capita and year. Blue countries spend more than 4000 euro and will be given two extra experts. Green countries spend more than 2000 euros and will be given one extra expert.

##

## Supplementary Figure 2. Nominated experts according to EU membership


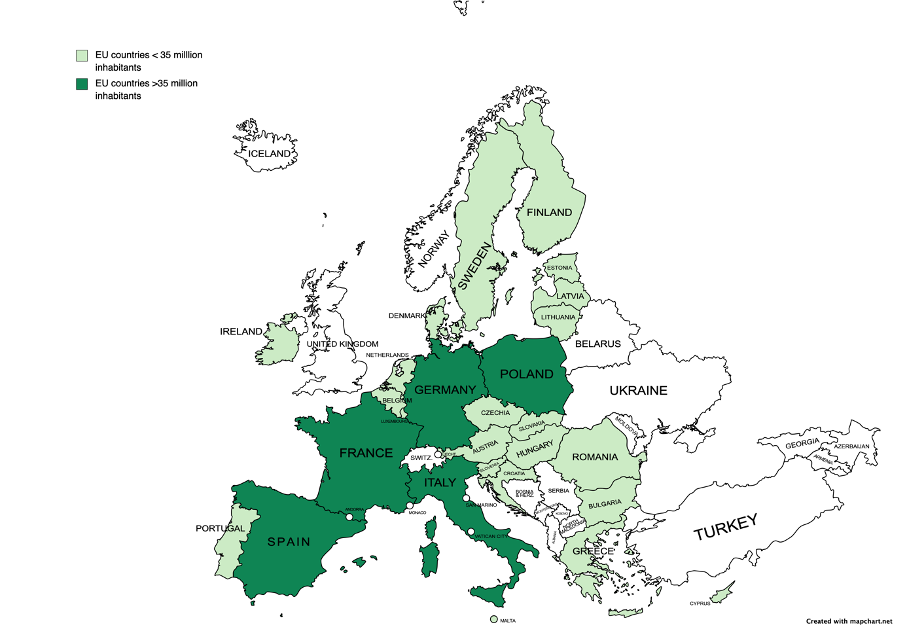


Supplementary Figure 2. EU countries are marked with green. They will be given one extra expert if they have less than 35 million inhabitants (light green) and two extra experts if they have more than 35 million inhabitants (dark green).

## Supplementary Figure 3. Total number of nominated experts per country


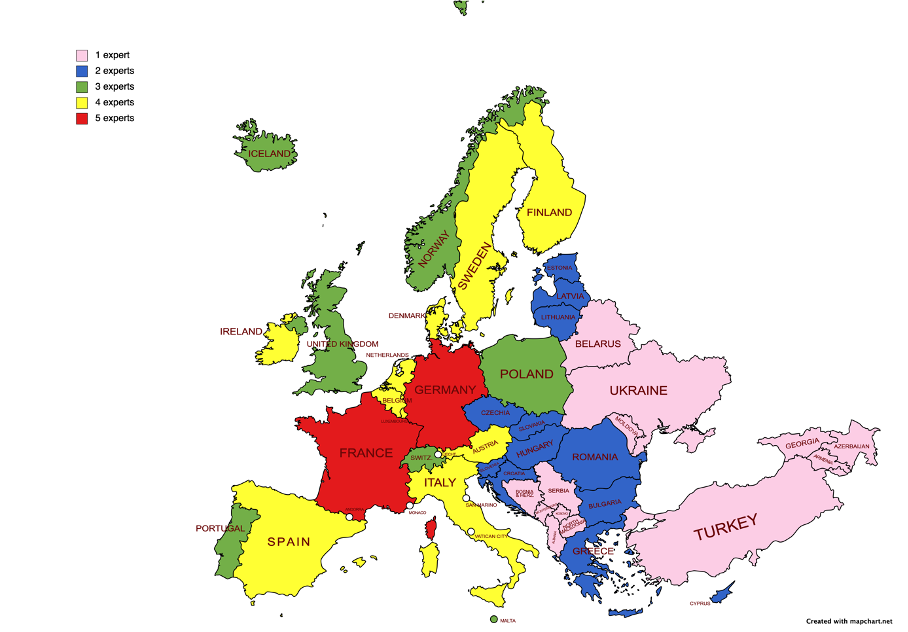


Supplementary Figure 3. Number of experts per country.

## Supplementary Table 1. Nominated experts per country and type

|  | **Plastic surgeon** | **Recon nurse** | **Patient rep** | **Total** |
| --- | --- | --- | --- | --- |
| **ESPRAS North** (1 delegate) |  |  |  |  |
| Denmark*^€€^ | 2 | 1 | 1 | **4** |
| Finland*^€€^ | 2 | 1 | 1 | **4** |
| Iceland^€€^ | 2 | 1 |  | **3** |
| Sweden*^€€^ | 2 | 1 | 1 | **4** |
| **ESPRAS west** (3 delegates) |  |  |  |  |
| Germany**^€€^ | 2 | 2 | 1 | **5** |
| Ireland*^€€^ | 2 | 1 | 1 | **4** |
| Luxemburg*^€€^ | 2 | 1 | 1 | **4** |
| Netherlands*^€€^ | 2 | 1 | 1 | **4** |
| Switzerland^€€^ | 2 | 1 |  | **3** |
| United Kingdom^€€^ | 2 | 1 |  | **3** |
| **ESPRAS South-west** (2 delegates**)** |  |  |  |  |
| Italy**^€^ | 2 | 1 | 1 | **4** |
| Portugal*^€^ | 2 | 1 |  | **3** |
| Malta* | 2 | 1 |  | **3** |
| Spain**^€^ | 2 | 1 | 1 | **4** |
| France**^€€^ | 2 | 2 | 1 | **5** |
| **ESPRAS Central and East** (2 delegates**)** |  |  |  |  |
| Austria*^€€^ | 2 | 1 | 1 | **4** |
| Belarus | 1 |  |  | **1** |
| Czechia* | 2 |  |  | **2** |
| Estonia* | 2 |  |  | **2** |
| Georgia | 1 |  |  | **1** |
| Hungary* | 2 |  |  | **2** |
| Latvia* | 2 |  |  | **2** |
| Lithuania* | 2 |  |  | **2** |
| Poland** | 2 | 1 |  | **3** |
| Slovakia* | 2 |  |  | **2** |
| **ESPRAS South-east** (2 delegates) |  |  |  |  |
| Armenia | 1 |  |  | **1** |
| Albania | 1 |  |  | **1** |
| Azerbaijan | 1 |  |  | **1** |
| Bulgaria* | 2 |  |  | **2** |
| Bosnia and Herzegovina | 1 |  |  | **1** |
| Croatia* | 2 |  |  | **2** |
| Cyprus* | 2 |  |  | **2** |
| Greece* | 2 |  |  | **2** |
| North Macedonia | 1 |  |  | **1** |
| Moldova | 1 |  |  | **1** |
| Romania* | 2 |  |  | **2** |
| Serbia | 1 |  |  | **1** |
| Slovenia* | 2 |  |  | **2** |
| Turkey | 1 |  |  | **1** |
| Ukraine | 1 |  |  | **1** |
| **Non-ESPRAS countries** |  |  |  |  |
| Belgium*^€€^ | 2 | 1 | 1 | **4** |
| Norway^€€^ | 2 | 1 |  | **3** |
| Kosovo | 1 |  |  | **1** |
| Montenegro | 1 |  |  | **1** |
| **TOTAL** | **75** | **21** | **12** | **108** |

Table S2:2. Experts per country

*One extra expert – EU country <35 million inhabitants

**Two extra experts – EU country >35 million inhabitants

€ One extra expert – spends >2000 euro per capita and year on healthcare

€€Two extra experts – spends >4000 euro per capita and year on healthcare

Grey marking: The countries can choose if they want to include a breast recon nurse or a patient rep depending on the organisation of the country

##

## Supplementary Table 2: Suggested quality indicators in pre-round and scores in Delphi round 1

All the suggested quality indicators in from the pre-round and the scores in Delphi round 1 are given in Table S4.1.

| **Suggested quality indicators in pre-round and scores in Delphi round 1** | **Total (n=43)** | | **Plastic surgeons (n=32)** | | **Patients (n=7)** | | **Reconstructive nurses (n=4)** | |
| --- | --- | --- | --- | --- | --- | --- | --- | --- |
|  | **Median** | **25th percentil** | **Median** | **25th percentil** | **Median** | **25th percentil** | **Median** | **25th percentil** |
| ***Access to and type of care*** |  |  |  |  |  |  |  |  |
| The proportion of patients who have access to immediate breast reconstruction | 5 | 5 | 5 | 4.5 | 5 | 5 | 5 | 4.75 |
| The proportion of patients who have access to immediate breast reconstruction before radiotherapy | 4 | 3 | 4 | 3 | 4 | 3 | 3 | 2 |
| The proportion of patients who are informed of the possibility of delayed breast reconstruction | 5 | 4 | 4 | 4 | 5 | 4.5 | 4.5 | 3.75 |
| The proportion of patients who are informed of the possibility of immediate breast reconstruction | 5 | 5 | 5 | 5 | 5 | 5 | 4.5 | 4 |
| The proportion of patients who are offered immediate breast reconstruction | 5 | 4 | 5 | 5 | 5 | 4 | 4.5 | 3.75 |
| The proportion of patients who are offered delayed breast reconstruction | 4 | 4 | 4 | 4 | 4 | 4 | 4.5 | 3.75 |
| The proportion of patients who are offered delayed autologous breast reconstruction | 4 | 3.25 | 4 | 4 | 4 | 3 | 3.5 | 3 |
| proportion of muscle flaps (that is. the frequency of TRAMs and LDs should be low) | 5 | 4 | 5 | 4 | 5 | 4.5 | 5 | 4.75 |
| The proportion of patients who are offered contralateral symmetrisation at the same time as the reconstruction on the cancer side | 4 | 4 | 4 | 3 | 4 | 4 | 4.5 | 3.75 |
| The proportion of patients who are offered areola tattoos in the same unit as the reconstruction is performed | 3 | 3 | 3 | 3 | 4 | 3 | 1.5 | 1 |
| The proportion of patients who receive their personal choice of reconstructive method | 4 | 4 | 4 | 3.5 | 5 | 5 | 5 | 4.75 |
| Whether equal access to breast reconstruction is offered in the country | 5 | 4 | 5 | 4 | 5 | 5 | 5 | 4.75 |
| The proportion of patients who are offered a choice of different implants | 3.5 | 2.25 | 3 | 2 | 5 | 4 | 3.5 | 2.75 |
| Ratio of implant-based to autologous delayed breast reconstruction | 4 | 3 | 4 | 3 | 4 | 3.25 | 3.5 | 3 |
| Ratio of implant-based to autologous immediate breast reconstruction | 4 | 3 | 4 | 3 | 5 | 4 | 3 | 2.75 |
| Ratio of mastectomies to mastectomies with single stage reconstruction | 4 | 3.25 | 4 | 4 | 4 | 3.5 | 3.5 | 3 |
| Ratio of immediate to delayed breast reconstruction | 4 | 4 | 5 | 4 | 4 | 4 | 4 | 4 |
| Frequency of breast-conserving surgery where a reconstructive surgeon performs volume replacement surgery (large perforator flaps) to avoid unnecessary mastectomy | 4 | 3.25 | 4 | 3 | 5 | 4 | 4 | 3.75 |
| Ratio of acellular dermal matrices (ADMs) vs. synthetic meshes | 3 | 2 | 3 | 1.5 | 3 | 3 | 3 | 2.5 |
| The proportion of patients who have access to pre-surgery patient education in group | 4 | 3 | 3 | 3 | 5 | 4.5 | 5 | 4.25 |
| The proportion of patients who have access to post-operative follow-up | 5 | 4.25 | 5 | 4 | 5 | 5 | 5 | 4.75 |
| The proportion of patients who receive preoperative counseling and support | 5 | 4 | 5 | 4 | 5 | 5 | 5 | 4.75 |
| The proportion of patients who have the possibility to take an active part in decision-making concerning breast reconstruction | 5 | 4 | 4 | 4 | 5 | 4.5 | 5 | 4.75 |
| The proportion of patients who have access to post-op physiotherapy focused on breast reconstruction patients | 4 | 3 | 4 | 3 | 5 | 4 | 4.5 | 4 |
| The proportion of patients who are shown photos of pre- and postoperative of different kinds results of different for types of women | 4 | 2.25 | 3 | 2 | 5 | 4.5 | 5 | 4.25 |
| The proportion of patients who have a contact nurse available pre- and postoperatively | 4 | 3.25 | 4 | 3 | 5 | 5 | 5 | 4.75 |
| The proportion of patients who have the option to meet a psychologist (tied to the team) | 4 | 3 | 4 | 3 | 5 | 4 | 3 | 2.75 |
| The proportion of patients who have the possibility of continuity and meeting the same healthcare staff in the team | 4 | 4 | 4 | 4 | 5 | 4 | 4 | 3 |
| The proportion of patients who have access to person-centered care | 4 | 3.25 | 4 | 3 | 5 | 5 | 4 | 3.75 |
| C | 4 | 4 | 5 | 4 | 4 | 4 | 3 | 3 |
| The proportion of nipple reconstructions | 4 | 3 | 4 | 3 | 5 | 4.5 | 3.5 | 2.5 |
| The proportion of different implant textures | 3 | 2 | 3 | 2 | 4 | 3.5 | 2.5 | 2 |
| The proportion of usage of PROMs for designing therapeutic strategies | 4 | 3 | 4 | 3.5 | 4 | 3 | 3 | 3 |
| Ratio of breast-conserving surgery +/- oncoplasty to mastectomy | 4 | 3 | 4 | 3 | 5 | 3.5 | 4 | 4 |
| Ratio of autologous flap-based reconstruction to total reconstruction using lipofilling | 3 | 3 | 3 | 2 | 4 | 3 | 3 | 3 |
| The proportion of units offering both implant-based and autologous breast reconstruction | 5 | 4 | 5 | 4 | 4 | 4 | 5 | 4.75 |
| The proportion of patients offered aesthetic flat chest procedure /bilateral aesthetic flat closure as an alternative to breast reconstruction | 3 | 2 | 3 | 2 | 4 | 3.5 | 4.5 | 3.75 |
| The proportion of uncompleted multi-stage breast reconstructions | 4 | 3 | 4 | 3 | 4 | 4 | 3.5 | 3 |
| Ratio of patients having breast reconstructions in urban to rural regions | 4 | 3 | 4 | 3 | 4 | 3 | 4 | 3.75 |
| The proportion of healthy older women who have access to breast reconstruction with all techniques | 4 | 4 | 4 | 4 | 5 | 4.5 | 3.5 | 2.75 |
| Ancillary | 4 | 4 | 5 | 4 | 4 | 3 | 4 | 3.75 |
| The proportion of units offering different flaps for autologous breast reconstruction | 4 | 3 | 4 | 3 | 4 | 3.25 | 4 | 3.75 |
| The proportion of women who have access to ancillary procedures and touch-ups | 4 | 4 | 4 | 3.5 | 4 | 4 | 4 | 3.75 |
| The proportion of patients who have access to both implant-based and autologous reconstruction | 5 | 4.25 | 5 | 4.5 | 5 | 4.5 | 5 | 4.75 |
| The proportion of patients who have (all parts of) their breast reconstruction covered by the public health care system | 5 | 4 | 5 | 4 | 5 | 5 | 5 | 4.5 |
| The proportion of patients who receive prophylactic antibiotics | 3 | 2 | 3 | 2 | 3 | 3 | 3 | 2.75 |
| Proportion of muscle flaps (that is. the frequency of TRAMs and LDs should be low) | 4 | 3 | 4 | 3 | 3 | 3 | 3.5 | 3 |
| The proportion of reconstructed patients who have significant risk factors for complications (radiotherapy. previous breast surgery. axillary dissection. smoking. high BMI >35. low BMI <19. co-morbidity. large volume breasts (>1200 ml?). | 4 | 3 | 4 | 3 | 4 | 4 | 3 | 3 |
| The proportion of cases in which decision aids or counselling tools are used pre-operatively | 4 | 3 | 3 | 3 | 4 | 4 | 3.5 | 2.75 |
| The proportion of women who are informed about how to prevent post-operative complications (eg. wound care. use of compression bandages. mobilisation. and anticoagulants) | 5 | 4 | 4 | 4 | 5 | 5 | 5 | 5 |
|  |  |  |  |  |  |  |  |  |
| ***Charachteristcs of the unit*** |  |  |  |  |  |  |  |  |
| A system for monitoring outcomes in the unit | 5 | 4 | 5 | 4 | 4 | 4 | 5 | 4.75 |
| Availability of acellular dermal matrices (ADMs) | 3 | 3 | 3 | 2 | 4 | 3.5 | 3 | 3 |
| Number of experienced surgeons in the unit | 5 | 4 | 5 | 4 | 5 | 4.5 | 5 | 5 |
| Number of immediate and delayed cases performed per year | 5 | 4 | 5 | 4 | 4 | 4 | 4 | 3.5 |
| Number of breast cancer cases per year in unit | 5 | 4 | 5 | 4 | 4 | 4 | 4 | 3.75 |
| Number of plastic surgeons specialised in microsurgery in the unit | 4 | 4 | 4 | 4 | 4 | 3.5 | 4.5 | 4 |
| Access to genetic testing and counselling if there is a family history | 5 | 5 | 5 | 5 | 5 | 5 | 5 | 5 |
| Application of Enhanced Recovery after Surgery (ERAS) protocols | 4 | 3 | 4 | 3 | 4 | 4 | 3.5 | 3 |
| Access to a psychologist particularly if considering risk reducing surgery | 4 | 3 | 4 | 3 | 5 | 4.5 | 3.5 | 2.75 |
| Access to a specialist nurse (specialised in breast reconstruction) | 5 | 4 | 4 | 3.5 | 5 | 5 | 4.5 | 3.75 |
| Access to written and/or video information regarding reconstructive options | 5 | 3.25 | 4 | 3 | 5 | 5 | 5 | 4.5 |
| Active research in the unit | 4 | 4 | 4 | 4 | 4 | 4 | 4 | 4 |
|  |  |  |  |  |  |  |  |  |
| ***Patient experiences and satisfaction*** |  |  |  |  |  |  |  |  |
| The proportion of patients who feel that their own choice was respected and central to the decision-making | 5 | 4 | 5 | 4 | 5 | 5 | 5 | 4.75 |
| Partner's evaluation of the psychological outcomes | 3 | 3 | 3 | 3 | 4 | 3 | 3 | 3 |
| Patient-reported satisfaction vs surgeon-reported satisfaction: How well do the surgeon and patient understand each other's expectations of what is a satisfactory result? | 5 | 3.25 | 4 | 3 | 5 | 5 | 4.5 | 4 |
| The proportion of patients who would have made the same choice if asked 1-3 years later. | 4 | 4 | 4 | 4 | 5 | 5 | 4 | 3.75 |
| The proportion of patients who have decisional regrets about reconstruction | 4 | 3.25 | 4 | 3.5 | 5 | 4.5 | 3.5 | 3 |
| The proportion of patients who are satisfied with the reconstruction | 5 | 4.25 | 5 | 4 | 5 | 5 | 5 | 4.75 |
| The proportion of patients who have restoration of lifestyle after mastectomy and reconstruction: In terms of resuming work. social life. sports activities. etc. | 5 | 4 | 5 | 4 | 5 | 5 | 5 | 4.75 |
| The proportion of patients who are satisfied with the shape | 5 | 4 | 5 | 4 | 5 | 4.5 | 4 | 3.75 |
| The proportion of patients who are satisfied with symmetry | 5 | 4 | 4 | 4 | 5 | 5 | 4 | 4 |
| The proportion of of patients who receive emotional support | 4 | 3 | 4 | 3 | 5 | 5 | 3.5 | 3 |
| The proportion of patients who feel they had enough time to think about reconstructive options | 4 | 4 | 4 | 4 | 5 | 5 | 4 | 3.75 |
| The proportion of patients who experience adequate pain management post-operatively | 4 | 4 | 4 | 3 | 5 | 5 | 4 | 4 |
| The proportion of patients who are aware of risks and benefits with different reconstructive options | 5 | 4 | 5 | 4 | 5 | 5 | 5 | 5 |
| The proportion of patients who have the possibility to discuss with other patients preoperatively | 4 | 3 | 3 | 3 | 5 | 4 | 4 | 3 |
| Partner's evaluation of the reconstructive outcomes | 3 | 2 | 3 | 2 | 4 | 2 | 2 | 2 |
| The proportion of patients who are treated like adults | 3.5 | 2 | 3 | 2 | 5 | 3.5 | 5 | 5 |
| The proportion of patients who are satisfied with the aesthetic outcome of the reconstruction | 5 | 4 | 5 | 4 | 5 | 5 | 4 | 4 |
| The proportion of patients who are satisfied with the size. shape. and feel of the reconstructed breast compared to their pre-surgery expectations | 5 | 4 | 5 | 4 | 5 | 5 | 4 | 3.75 |
| The proportion of patients who are satisfied with the freedom of movement and comfort in the reconstructed area | 4 | 4 | 4 | 4 | 5 | 5 | 4 | 4 |
| The proportion of patients who are satisfied with the healthcare staff's clarity. empathy. and support in relation to decision-making about the type of reconstruction | 5 | 4 | 4 | 4 | 5 | 5 | 4.5 | 4 |
| Psychosocial impact of the reconstruction over time | 4.5 | 4 | 4 | 3 | 5 | 5 | 4.5 | 4 |
| The proportion of patients who are satisfied with sensibility | 4 | 3 | 3 | 3 | 5 | 4.5 | 3.5 | 2.75 |
| The proportion of patients who are satisfied with feel-to-touch | 4 | 3.25 | 4 | 3 | 5 | 5 | 4 | 3.75 |
| The proportion of patients who are wearing a bra comfortably | 4 | 4 | 4 | 3.5 | 5 | 4 | 4 | 4 |
| The proportion of patients who feel free to wear a swim suit | 4 | 3 | 4 | 3 | 5 | 3.5 | 4 | 3.75 |
|  |  |  |  |  |  |  |  |  |
| **Surgical outcomes and complications** |  |  |  |  |  |  |  |  |
| Frequency of minor complications | 3 | 3 | 3 | 3 | 3 | 3 | 3.5 | 3 |
| Frequency of major complications | 5 | 5 | 5 | 5 | 5 | 5 | 4.5 | 3.5 |
| Operation time | 4 | 3 | 4 | 2.5 | 4 | 3.5 | 4 | 4 |
| Frequency of complications needding revision surgery according to Calvin Dindo? | 4 | 4 | 4 | 4 | 4 | 4 | 3 | 3 |
| Frequency of re-admissions | 4 | 4 | 4 | 4 | 4 | 4 | 3.5 | 2.75 |
| Length of hospital stay | 4 | 3 | 4 | 3 | 4 | 3 | 4 | 3.75 |
| Frequency of loss of autologous flaps | 5 | 4 | 5 | 5 | 4 | 3.5 | 4 | 4 |
| Recovery time (time to returning to work. time to time free. time to ability to perform daily tasks at the same physical level as pre-surgery) | 4 | 4 | 4 | 4 | 5 | 4.5 | 4 | 4 |
| Frequency of complications the first 30 days | 5 | 4 | 5 | 4 | 5 | 4 | 4.5 | 3.5 |
| Frequency of complications after 30 days | 4 | 4 | 4 | 4 | 5 | 4 | 3.5 | 2.75 |
| Frequency of complications after 60 days | 4 | 3 | 4 | 3 | 5 | 4 | 4 | 3.5 |
| Frequency of skin necrosis | 5 | 4 | 5 | 4 | 5 | 4.5 | 4 | 3.5 |
| Frequency of secondary corrections | 4 | 3 | 4 | 3 | 4 | 3.5 | 3.5 | 3 |
| Frequency of tertiary corrections | 4 | 3 | 4 | 3 | 5 | 3.5 | 3.5 | 2.75 |
| Frequency of infections | 5 | 4 | 5 | 4 | 5 | 4.5 | 4.5 | 3.5 |
| The proportion of patients receiving therapeutic antibiotics | 4 | 3 | 4 | 2 | 4 | 4 | 3 | 3 |
| Frequency of fat necrosis | 4 | 3 | 4 | 3 | 5 | 4 | 4 | 4 |
| Frequency of donor site complications | 4 | 4 | 4 | 3.5 | 5 | 4 | 4 | 3.75 |
| Ischaemia time | 3 | 2 | 3 | 2 | 4.5 | 4 | 4 | 4 |
| Frequency of patients who have shoulder dysfunction after immediate breast reconstruction | 4 | 3 | 4 | 3 | 5 | 4.5 | 5 | 4 |
| Re-operation rates | 5 | 4 | 5 | 4 | 5 | 4.25 | 5 | 4.5 |
| Frequency of clear margins | 5 | 4 | 5 | 5 | 4 | 4 | 4.5 | 3.75 |
| Frequency of partial flap loss | 4 | 4 | 4 | 4 | 5 | 4 | 4 | 4 |
| Frequency of implant loss | 5 | 4 | 5 | 4.5 | 5 | 5 | 4 | 4 |
| Frequency of animation deformity | 4 | 3 | 4 | 3 | 4 | 4 | 4 | 3.5 |
| Longevity/durability of the reconstruction | 5 | 4 | 4 | 4 | 5 | 4.5 | 5 | 4.5 |
| Frequency of lymphoedema | 5 | 3.25 | 5 | 3 | 5 | 5 | 4.5 | 3.75 |
| Frequency of irradiated reconstruction (radiation post-reconstruction) | 4 | 4 | 4 | 4 | 4 | 4 | 3 | 3 |
| Frequency of BIA-ALCL | 4 | 3 | 4 | 3 | 4 | 3.5 | 3.5 | 3.25 |
| Frequency of re-operations after implant-based reconstruction | 5 | 4 | 5 | 4 | 5 | 4 | 4 | 2.75 |
| Frequency of re-operations after irradiated implant-based reconstruction | 5 | 4 | 5 | 4 | 5 | 4.5 | 4 | 3 |
| Frequency of conversion to other reconstructive techniques | 4 | 3 | 4 | 3 | 4 | 3.5 | 4 | 3.5 |
| Frequency of cosmetic correction | 4 | 3 | 4 | 3 | 4 | 3.5 | 4 | 3.75 |
| Frequency of haematoma | 4 | 3 | 4 | 3 | 4 | 4 | 3.5 | 3 |
| Frequency of seroma | 4 | 3 | 4 | 3 | 5 | 3 | 3.5 | 3 |
| Frequency of capsular contracture | 4 | 4 | 4 | 4 | 5 | 4.5 | 4 | 3.75 |
|  |  |  |  |  |  |  |  |  |
| ***Waiting times and treatment timelines*** |  |  |  |  |  |  |  |  |
| Waiting time to a consultation for delayed breast reconstruction | 4 | 3 | 4 | 3 | 4 | 4 | 4 | 4 |
| Waiting time for single stage reconstruction | 4 | 3.25 | 4 | 3.5 | 5 | 3.5 | 4 | 3.75 |
| Waiting time for second stage of reconstruction | 4 | 3 | 4 | 3 | 4 | 3.5 | 4 | 3.75 |
| Waiting time for delayed breast reconstruction | 4 | 3 | 4 | 3 | 5 | 4.5 | 4 | 4 |
| Waiting time for delayed reconstruction | 4 | 3 | 4 | 3 | 4 | 4 | 4 | 4 |
| Waiting time from the initial biopsy to surgery with immediate reconstruction | 5 | 4 | 5 | 4 | 5 | 4.5 | 5 | 4.75 |
| Waiting time from the end of the last cycle of neoadjuvant chemotherapy to surgery with immediate reconstruction | 5 | 4 | 5 | 4 | 5 | 4.5 | 4.5 | 4 |
| Time to start of adjuvant therapy if the latter is indicated | 5 | 4 | 5 | 4 | 4 | 4 | 4 | 4 |
| Time from mastectomy to reconstruction | 4 | 3.25 | 4 | 3 | 5 | 4 | 4 | 4 |
| Time between the request of reconstruction and the surgery | 4 | 3 | 3 | 3 | 4 | 4 | 4 | 4 |
| Proportion of patients who have completed the breast reconstruction in a year or less | 4 | 3 | 4 | 3 | 4 | 4 | 4 | 4 |
|  |  |  |  |  |  |  |  |  |
| ***Multidisciplinary collaborations*** |  |  |  |  |  |  |  |  |
| The proportion of cases where plastic surgeons are routine members of the breast team | 5 | 4 | 5 | 4.5 | 5 | 4.5 | 4 | 3.75 |
| The proportion of cases where plastic surgeons are involved in breast reconstruction | 5 | 5 | 5 | 5 | 5 | 5 | 5 | 4.75 |
| The proportion of cases where individualised treatment planning is performed | 5 | 4 | 5 | 4 | 5 | 5 | 5 | 4.75 |
| The proportion of cases where multidisciplinary planning is performed | 5 | 4 | 5 | 4 | 5 | 5 | 5 | 4.75 |
| The proportion of cases where MDT meetings. which always includes a plastic surgeon. are performed before and after surgery | 5 | 4 | 5 | 4 | 5 | 5 | 4 | 3.75 |
| Access to multidisciplinary breast unit (Breast Surgeon. Plastic Surgeon. Oncologist. Radiotherapist. Radiology. Psychologist) | 5 | 5 | 5 | 5 | 5 | 5 | 5 | 5 |

Supplementary Table 2. Suggested quality indicators in the pre-round and their scores in the first Delphi round. Indicators going through to the second Delphi round are marked in yellow if they reached consensus in the total group and in orange if they reached consensus in one of the subgroup.

## Supplementary Table 3: Discarded quality indicators in the different Delphi rounds

| **Discarded quality indicators** | **Round 1 (n=43)** |  |  |  |
| --- | --- | --- | --- | --- |
|  | **Median** | **25th percentil** |  |  |
| **Discarded in Delphi round 1** |  |  |  |  |
| The proportion of patients who have access to immediate breast reconstruction before radiotherapy | 4 | 3 |  |  |
| The proportion of patients who are offered delayed autologous breast reconstruction | 4 | 3.25 |  |  |
| The proportion of patients who are offered areola tattoos in the same unit as the reconstruction is performed | 3 | 3 |  |  |
| The proportion of patients who are offered a choice of different implants | 3.5 | 2.25 |  |  |
| Ratio of implant-based to autologous delayed breast reconstruction | 4 | 3 |  |  |
| Ratio of implant-based to autologous immediate breast reconstruction | 4 | 3 |  |  |
| Ratio of mastectomies to mastectomies with single stage reconstruction | 4 | 3.25 |  |  |
| Frequency of breast-conserving surgery where a reconstructive surgeon performs volume replacement surgery (large perforator flaps) to avoid unnecessary mastectomy | 4 | 3.25 |  |  |
| Ratio of acellular dermal matrices (ADMs) vs. synthetic meshes | 3 | 2 |  |  |
| The proportion of patients who have the option to meet a psychologist (tied to the team) | 4 | 3 |  |  |
| The proportion of patients who have access to person-centered care | 4 | 3.25 |  |  |
| The proportion of nipple reconstructions | 4 | 3 |  |  |
| The proportion of different implant textures | 3 | 2 |  |  |
| The proportion of usage of PROMs for designing therapeutic strategies | 4 | 3 |  |  |
| Ratio of breast-conserving surgery +/- oncoplasty to mastectomy | 4 | 3 |  |  |
| Ratio of autologous flap-based reconstruction to total reconstruction using lipofilling | 3 | 3 |  |  |
| The proportion of patients offered aesthetic flat chest procedure /bilateral aesthetic flat closure as an alternative to breast reconstruction | 3 | 2 |  |  |
| The proportion of uncompleted multi-stage breast reconstructions | 4 | 3 |  |  |
| Ratio of patients having breast reconstructions in urban to rural regions | 4 | 3 |  |  |
| The proportion of units offering different flaps for autologous breast reconstruction | 4 | 3 |  |  |
| The proportion of patients who receive prophylactic antibiotics | 3 | 2 |  |  |
| Proportion of muscle flaps (that is. the frequency of TRAMs and LDs should be low) | 4 | 3 |  |  |
| The proportion of reconstructed patients who have significant risk factors for complications (radiotherapy. previous breast surgery. axillary dissection. smoking. high BMI >35. low BMI <19. co-morbidity. large volume breasts (>1200 ml?). | 4 | 3 |  |  |
| The proportion of cases in which decision aids or counselling tools are used pre-operatively | 4 | 3 |  |  |
| Availability of acellular dermal matrices (ADMs) | 3 | 3 |  |  |
| Application of Enhanced Recovery after Surgery (ERAS) protocols | 4 | 3 |  |  |
| Access to a psychologist particularly if considering risk reducing surgery | 4 | 3 |  |  |
| Access to written and/or video information regarding reconstructive options | 5 | 3.25 |  |  |
| Partner's evaluation of the psychological outcomes | 3 | 3 |  |  |
|  |  |  | **Round 2 (n=43)** | |
| **Discarded in Delphi round 2** |  |  | **Agreement** |  |
| The proportion of who receive their personal choice of reconstructive method | 4 | 4 | 64% |  |
| Acess to volume displacement oncoplastic surgery (Clough I and II, intraglandular flap) | 4 | 4 | 50% |  |
| Acess to ancillary procedures and touch-ups | 4 | 4 | 38% |  |
| Acess to reconstruction specifically after nipple-sparing mastectomies | 4 | 4 | 52% |  |
| Acess to reconstruction in healthy older women | 5 | 4 | 41% |  |
| Acess to contralteral symemtrisation at the reconstruction on the cancer side | 4 | 4 | 60% |  |
| How well do the surgeon and patient understand each other's expectations of what is a satisfactory result? | 5 | 3.25 | 65% |  |
| The proportion of patients who feel they had enough time to think about reconstructive options | 4 | 4 | 67% |  |
| The proportion of patients who receive preoperative counseling and support | 5 | 4 | 63% |  |
| The proportion of patients who have the possibility to take an active part in decision-making concerning breast reconstruction | 5 | 4 | 45% |  |
| The proportion of patients who feel that their own choice was respected and central to the decision-making | 5 | 4 | 55% |  |
| Axcess to pre-surgical patient education in the group | 4 | 3 | 40% |  |
| The proportion of women who have demonstration of photos | 4 | 2.25 | 50% |  |
| The proportion of patients who have the possibility of continuity and meeting the same healthcare staff in the team | 4 | 4 | 56% |  |
| The proportion of patients who have a contact nurse available pre- and postoperatively | 4 | 3.25 | 67% |  |
| Access to a specialist nurse (specialised in breast reconstruction) | 5 | 4 | 63% |  |
| Number of plastic surgeons specialised in microsurgery in the unit | 4 | 4 | 67% |  |
| Patient satisfaction with wearing a bra comfortably | 4 | 4 | 46% |  |
| Patient satisfaction with shape (re-phrased from The proportion of patients who are satisfied with the size. shape. and feel of the reconstructed breast compared to their pre-surgery expectations) | 5 | 4 | 54% |  |
| Patient satisfaction with how the reconstrcted breast feels to touch (The proportion of patients who are satisfied with the size. shape. and feel of the reconstructed breast compared to their pre-surgery expectations) | 5 | 4 | 42% |  |
| Patient satisfaction with sensibility (The proportion of patients who are satisfied with the size. shape. and feel of the reconstructed breast compared to their pre-surgery expectations) | 5 | 4 | 32% |  |
| Ischemia time | 3 | 2 | 39% |  |
| Frequency of re-operations after implant-based reconstruction | 5 | 4 | 49% |  |
| Frequency of re-operation after irradated implant-based reconstruction | 5 | 4 | 42% |  |
| Frequency of partial flap loss | 4 | 4 | 47% |  |
| Frequency of donor site complications | 4 | 4 | 58% |  |
| Frequency of shoulder dysfunction after immediate breast reconstruction | 4 | 3 | 49% |  |
| Waiting time to a consultation for a delayed breast reconstruction | 4 | 3 | 23% |  |
| Waiting time for delayed breast reconstruction | 4 | 3 | 67% |  |
| Waiting time from the end of last cycle of neoadjuvant chemotherapy to surgery with immediate reconstuction | 5 | 4 | 47% |  |
| Waiting time from mastectomy to reconstruction | 4 | 3.25 | 49% |  |
| Time between the request of reconstruction and the surgery | 4 | 3 | 47% |  |
| Time to start of adjuvant therapy if the latter is indicated | 5 | 4 | 51% |  |
| The proportion of cases where plastic surgeons are routine members of the breast team | 5 | 4 | 63% |  |
| The proportion of cases where plastic surgeons are involved in breast reconstruction | 5 | 5 | 49% |  |
| The proportion of cases where multidisciplinary planning is performed | 5 | 4 | 63% |  |
| The proportion of cases where multidisciplart team (MDT) meetings, which alwaus include a plastic surgeon is performed | 5 | 4 | 68% |  |
| The proportion of patients who are offered delayed breast reconstruction | 4 | 4 | 68% |  |
|  |  |  |  | **Round 3 (n=43)** |
| **Discarded in Delphi round 3** |  |  |  | **Agreement** |
| The proportion of patients that are offered delayed breast reconstruction | 4 | 4 | 74%* | 74% |
| Ratio of immediate to delayed breast reconstructions | 4 | 4 | 72%* | 70% |
| The proportion of patients that have (all parts of) their breast reconstruction convered by the public health care system | 5 | 4 | 74%* | 70% |

Supplementary Table 3. Discarded quality indicators in the different Delphi rounds. *= Quality indicators that almost reached consensus (70.0-74.9%) in round 2 and were given a second chance in round 3.

# Supplementary Appendices

## Supplementary Appendix 1: Delphistar checklist


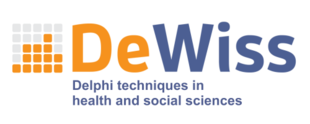

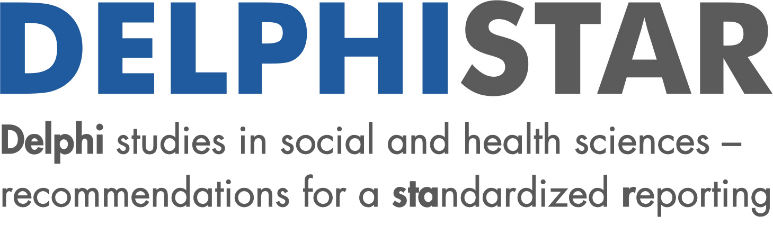

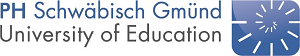


**Delphi studies in social and health sciences – recommendations for an interdisciplinary standardized reporting (DELPHISTAR).**

From: Niederberger, M., Schifano, J., Deckert, S., Hirt, J., Homberg, A., Köberich, S., Kuhn, R., Rommel, A., Sonnberger, M. & the DEWISS network (2024). Delphi studies in social and health sciences—Recommendations for an interdisciplinary standardized reporting (DELPHISTAR). Results of a Delphi study. *PLoS ONE 19(8):* e0304651. <https://doi.org/10.1371/journal.pone.0304651>

| **Topic** | **Section** | **Item** | **Checklist Item** | **Location where item is reported** | **Exemplary wording** |
| --- | --- | --- | --- | --- | --- |
| **I**  **Title and Abstract** |  | 1 | Identification as a Delphi study in the title | Title | What is a public health intervention? Results of a Delphi study. |
|  |  | 2 | Identification as a Delphi study in the abstract | Abstract | A Delphi study was selected to answer the research question. |
|  |  | 3 | Structured abstract | Abstract | e.g., background, method, results and discussion |
| **II**  **Context** | **Formal** | 4 | Information about the sources of funding | Title page, p. 17 | The Delphi study was funded by [SOURCE]. |
|  |  | 5 | Information about the team of authors and/or researchers (e.g., discipline, institution) | Title page, Supplement 2 | The Delphi study was conducted by an interdisciplinary team with representatives from medicine, public health, and health promotion. |
|  |  | 6 | Information about method consulting | p. 5 | The study group was advised by experts from [INSTITUTION] regarding statistics.  Or:  No consulting in regard to method took place. |
|  |  | 7 | Information about the project background | pp. 2-3  pp. 3-4 | The Delphi study was part of a mixed-methods study on [AIM]. |
|  |  | 8 | Information about the study protocol | pp. 3-4 | The study protocol is available at [LINK]. |
|  | **Content** | 9 | Justification of the chosen method (Delphi) to answer the research question | p. 5 | The Delphi method is suitable for answering the research question because it systematically gathers the judgments of different expert groups and can identity agreement and disagreement. |
|  |  | 10 | Aim of the Delphi study (e.g., consensus, forecasting) | p. 3 | The aim of the Delphi study is to find consensus on criteria to define a public health intervention. |
| **III**  **Method** | **Body & Integration of knowledge** | 11 | Identification and elucidation of relevant expertise, spheres of experience, and perspectives (e.g., theory, practice, affected groups, disciplines) | pp. 4-5, Supplement 3, Table 1, Figures 1-2 | The experts represent the sciences and clinical practice because [REASON]. |
|  |  | 12 | Handling of knowledge, expertise and perspectives which are missing or have been deliberately not integrated | pp. 4-5, Supplement 3, Table 1, Figures 1-2 | If it is not possible to recruit experts specialized in [AREA], this will be openly communicated to the other experts during the Delphi study. |
|  |  | 13 | Basic definition of expert^1^ | Table 1 | A person who has been active in the area for at least [NUMBER] years is considered to be an expert. |
|  | **Delphi variant and modifications** | 14 | Identification of the type of Delphi variant and potential modifications (e.g., classic Delphi, real-time Delphi, group Delphi) | pp. 5-6 | A classic Delphi study was used [LITERATURE REFERENCE]. |
|  |  | 15 | Justification of the Delphi variant and modifications, including during the Delphi study, if applicable | pp. 5-9 | If the willingness to participate clearly decreases between the first and second round, a third round will not be held. |
|  | **Sample of experts** | 16 | Selection criteria for the experts (per round, per expert group if applicable) | Table 1, Supplement 3 | All of the experts who met the definition were invited to the first round.  All of the experts who completed the previous round were invited to participate in the subsequent round. |
|  |  | 17 | Identification of the experts | pp. 4-5 | The experts were identified based on publications in [DATABASE]. |
|  |  | 18 | Information about recruiting and any subsequent recruiting of experts | pp. 4-5, Figure 2 | The experts were informed about the Delphi study and invited to participate. |
|  | **Survey** | 19 | Elucidation of the content development for the questionnaire^2^ | Figure 3, p. 7 | The questionnaire was developed based on the results of systematic reviews [LITERATURE REFERENCE]. |
|  |  | 20 | Description of the questionnaire (content and structure) | pp. 7-9 | The questionnaire was divided into three segments on [TOPICS]. The statements made in the questionnaire were evaluated using standardized items, with the option to comment in free-text boxes. |
|  | **Delphi rounds** | 21 | Number of Delphi rounds | pp. 7-9 | Three Delphi rounds were held. |
|  |  | 22 | Information about the aims of the individual Delphi rounds | pp. 7-9 | The first Delphi round focused on exploring relevant aspects. These aspects were then presented to the experts in the second Delphi round for standardized evaluation. |
|  |  | 23 | Disclosure and justification of the criterion for discontinuation | pp. 5-7 | The number of rounds was defined in advance to be a maximum of three rounds. |
|  | **Feedback** | 24 | Information about what data was reported back per round | pp. 5-7 | In terms of feedback, we shared the statistical results plus the summary of the open responses. |
|  |  | 25 | Information on how the results of the previous Delphi round were fed back to the experts surveyed (e.g., via frequencies, mean values, measures of dispersion, listing of comments) | pp. 5-7 | Mean values, standard deviations and percentage frequency distributions were reported. |
|  |  | 26 | Information on whether feedback was differentiated by specific groups (e.g., by field of expertise, institutional affiliation) | pp. 5-7 | The feedback was aggregated across all expert groups. |
|  |  | 27 | Information about how dissent and unclear results were handled | pp. 5-7 | The results showing dissent were presented again for evaluation in the next Delphi round. |
|  | **Data analysis** | 28 | Disclosure of the quantitative and qualitative analytical strategy | pp. 5-9 | The quantitative items were descriptively analyzed. The open-ended items were analyzed using thematic analysis [LITERATURE REFERENCE]. |
|  |  | 29 | Definition and measurement of consensus | pp. 5-9 | Consensus was defined as percentage agreement, meaning that agreement was assumed if at least 80% of the respondents agreed on an item. |
|  |  | 30 | Information on group-specific analysis or weighting of experts (e.g., theory vs. practice, discipline-specific analysis) | pp. 5-9 | In the analysis, the mean values for percent agreement are weighted for each expert group in terms of the number of group members. |
| **IV**  **Results** | **Delphi process** | 31 | Illustration of the Delphi study (e.g., in a flow chart) | Figure 3 | A summary of the Delphi study is illustrated in a flow chart (Figure 1). |
|  |  | 32 | Information about special aspects during the Delphi study (e.g., deviations from the intended approach with justification) | p. 8 | During the Delphi study the political discussion mentioned climate change and the effects on health. It is possible that this influenced the experts' responses. |
|  |  | 33 | Number of experts per round (both invited and participating) | Figure 2 | The number of experts participating in the first Delphi round was [NUMBER], and the number of experts in the second round was [NUMBER]. This corresponds to a response rate of [NUMBER]% in the first round and [NUMBER]% in the second round. |
|  | **Results** | 34 | Presentation of the results for each Delphi round and the final results | Table 2, Supplement, pp. 9-10, Supplements 4-6 | In the first Delphi round [NUMBER]% of the experts agreed, in the second [NUMBER]%, and in the third [NUMBER]%. |
| **V Discussion** | **Quality of findings** | 35 | Highlighting the findings from the Delphi study | p. 10 | The central findings can be summarized as follows: [STATE FINDINGS]. |
|  |  | 36 | Validity of the results (e.g., transferability of the findings) | pp. 12-13 | The results are not transferable to other countries due to different legal regulations. |
|  |  | 37 | Reliability of the results (e.g., split half, inter-rater reliability) | pp. 12-13 | The responses in the free-text comments were analyzed by two independent reviewers [SPECIFY]. |
|  |  | 38 | Reflection on potential limitations (e.g., number of experts, response bias) | pp. 12-13 | The results are to be viewed critically with regard to the composition of the panel because [REASONS]. |

^1^ “Experts” are the participants; these can be people from academia, practice, or representatives of lived experience (e.g., patients, family members).

^2^ The term “questionnaire” stands for the survey instrument regardless of whether quantitative or qualitative items are integrated or weighted.

## Supplementary appendix 2: Steering committee

Heads of the steering committee:

Professor Anna Elander, MD, PhD, board-certified plastic surgeon. Secretary General of the European Society of Plastic, Reconstructive and Aesthetic Surgery (ESPRAS) when the study was initiated. President of the Swedish Society of Plastic Surgeons (2024-).

Professor Riccardo Giunta, MD, PhD, board-certified plastic surgeon. President of ESPRAS when the study was initiated.

Principal investigators:

Professor Emma Hansson, MD, PhD, board-certified plastic surgeon. Former president of the Swedish Society of Plastic Surgeons (2018-2022).

Associate Professor Nicholas Moellhoff, MD, PhD, board-certified plastic surgeon.

Other members of the steering committee:

Mr Mark Henley, MD, board-certified plastic surgeon. President of ESPRAS when the study was concluded.

Ms. Ruth Waters, MD, board-certified plastic surgeon. President of the British Association of Plastic Surgeons when the study was initiated.

Professor Lisbet Rosenkrantz Hölmich, MD, PhD, board-certified plastic surgeon.

Associate Professor Ilkka Kaartinen, MD, PhD, board-certified plastic surgeon.

Ms Alexandra Uusimäkki, patient representative.

Mrs Susanne Ahlstedt Karlsson, RN, PhD.

**Supplementary Figures and Tables**

**References**
